# T3SS-Independent Uptake of the Short-Trip Toxin-Related Recombinant NleC Effector of Enteropathogenic *Escherichia coli* Leads to NF-κB p65 Cleavage

**DOI:** 10.3389/fcimb.2017.00119

**Published:** 2017-04-13

**Authors:** Anne-Sophie Stolle, Stefanie Norkowski, Britta Körner, Jürgen Schmitz, Lena Lüken, Maj Frankenberg, Christian Rüter, M. Alexander Schmidt

**Affiliations:** ^1^Institute of Infectiology, Center for Molecular Biology of Inflammation, University of MünsterMünster, Germany; ^2^Institute of Experimental Pathology, Center for Molecular Biology of Inflammation, University of MünsterMünster, Germany

**Keywords:** T3SS effectors, NleC, A-B toxins, EPEC, endocytosis, NF-κB signaling, endosomal escape

## Abstract

Effector proteins secreted by the type 3 secretion system (T3SS) of pathogenic bacteria have been shown to precisely modulate important signaling cascades of the host for the benefit of the pathogens. Among others, the non-LEE encoded T3SS effector protein NleC of enteropathogenic *Escherichia coli* (EPEC) is a Zn-dependent metalloprotease and suppresses innate immune responses by directly targeting the NF-κB signaling pathway. Many pathogenic bacteria release potent bacterial toxins of the A-B type, which—in contrast to the direct cytoplasmic injection of T3SS effector proteins—are released first into the environment. In this study, we found that NleC displays characteristics of bacterial A-B toxins, when applied to eukaryotic cells as a recombinant protein. Although lacking a B subunit, that typically mediates the uptake of toxins, recombinant NleC (rNleC) induces endocytosis via lipid rafts and follows the endosomal-lysosomal pathway. The conformation of rNleC is altered by low pH to facilitate its escape from acidified endosomes. This is reminiscent of the homologous A-B toxin AIP56 of the fish pathogen *Photobacterium damselae piscicida* (*Phdp*). The recombinant protease NleC is functional inside eukaryotic cells and cleaves p65 of the NF-κB pathway. Here, we describe the endocytic uptake mechanism of rNleC, characterize its intracellular trafficking and demonstrate that its specific activity of cleaving p65 requires activation of host cells e.g., by IL1β. Further, we propose an evolutionary link between some T3SS effector proteins and bacterial toxins from apparently unrelated bacteria. In summary, these properties might suggest rNleC as an interesting candidate for future applications as a potential therapeutic against immune disorders.

## Introduction

Enteropathogenic *Escherichia coli* (EPEC) cause severe and persistent intestinal infections, which can result in life threatening diarrhea in children. An important mechanism of bacterial pathogens, which promotes survival and hence results in prolonged infections, is the injection of effector proteins directly into host cells by intriguing nanomachines, such as the type 3 secretion system (T3SS). Several distinct T3SS-injected effector proteins target the host's immune responses by suppressing phagocytosis and/or the expression of pro-inflammatory cytokines (e.g., Dean et al., 2005; Dean and Kenny, 2009; Jayamani and Mylonakis, 2014; Reddick and Alto, 2014; Asrat et al., 2015; Ratner et al., 2016; Pearson et al., 2017). The pathogenicity island “locus of enterocyte effacement (LEE)” harbors the genes encoding the T3SS and also several effectors, which are largely responsible for inducing the “attaching and effacing (A/E)” phenotype observed during EPEC infections (McDaniel et al., 1995; Frankel et al., 1998). In contrast, the “non-LEE encoded effectors (Nle)” mostly target and modulate important signaling cascades (Dean et al., 2005; Dean and Kenny, 2009; Raymond et al., 2013; Jayamani and Mylonakis, 2014; Santos and Finlay, 2015; Yen et al., 2016), such as the Map kinase-signaling or NF-κB signaling cascades. These signaling cascades are central for the orchestration of several crucial host cell functions, such as proliferation, differentiation, apoptosis, and pro-inflammatory cytokine expression and therefore need to be precisely regulated (Baeuerle, 1998; Pahl, 1999; Karin et al., 2002; Perkins, 2004; Hayden and Ghosh, 2012; Le Negrate, 2012; Johannessen et al., 2013; Cildir et al., 2016) but are also important targets for the concerted activities of a number of different effector proteins of bacterial pathogens. Nle effector proteins of EPEC exhibit different enzymatic functions and undermine cellular responses by modulating essential signaling cascades at different levels (Rüter and Hardwidge, 2014). NleE is a cysteine methyltransferase targeting TAB2 and TAB3 (Zhang et al., 2011), whereas NleH binds to the NF-κB specifier RPS3 thereby inhibiting the translocation of RPS3 into the nucleus (Gao et al., 2009). NleC is a T3SS-dependent Zn-metalloprotease that cleaves the NF-κB subunit p65 and thus inhibits the translocation of NF-κB subunits into the nucleus (Yen et al., 2010; Baruch et al., 2011; Mühlen et al., 2011; Pearson et al., 2011). Due to the synergy of different effector protein activities, NF-κB signaling is almost completely shut down. Consequently, pro-inflammatory cytokine expression increases only slightly which results in a dampening of overall immune responses. This facilitates EPEC replication and promotes prolonged bacterial infection (Pearson et al., 2011).

Recently, ours and other laboratories have identified several virulence proteins of different bacterial pathogens including the T3SS-dependent effector proteins YopM from *Yersinia enterocolitica* (Rüter et al., 2010), SspH1 from *Salmonella enterica* serovar Typhimurium (Lubos et al., 2014), or the Tir protein of enteropathogenic *E. coli* (Michgehl et al., 2006) and TcpC from uropathogenic *E. coli* (UPEC) (Cirl et al., 2008; Yadav et al., 2010) that are able to translocate autonomously across eukaryotic membranes without a need for a T3SS or any other bacterial factor. These T3SS-associated effector proteins exhibit properties of cell-penetrating peptides (CPPs) and have been proposed to represent a novel class of “cell-penetrating effectors” or CPEs (Rüter et al., 2010; Yadav et al., 2010; Snyder et al., 2013; Lubos et al., 2014). Although the relevance of type-3-secretion-independent translocation during bacterial infections has not been unraveled yet, these findings prompted the development of innovative strategies of applying CPEs as self-delivering therapeutics against autoimmune diseases. The plethora of effectors, which have evolved and specialized during evolution, can thus be addressed as a “tool-pool” to precisely target and effectively modulate central signaling pathways of the host, thereby identifying potential bacteria-derived candidates for the development of therapeutic biologics (Rüter and Hardwidge, 2014; Rüter and Schmidt, 2017).

In this study, we characterized the interaction of the T3SS non-LEE encoded effector protein NleC as a recombinant isolated protein with host cells. We demonstrated that recombinant NleC (rNleC) can enter eukaryotic cells autonomously via endocytosis followed by endosomal escape. Subsequently, rNleC reaches its intracellular target p65 and partially cleaves the NF-κB p65 subunit requiring an activation of NF-κB signaling. *In silico* analyzes have suggested the presence of two potential protein transduction domains (PTDs) in NleC. Yet, these predicted PTDs were found not to be able to mediate the uptake of rNleC in host cells and, furthermore, failed in cargo transport. Hence, the recombinant T3SS effector NleC does not qualify as a CPE. Interestingly, we found that the mechanism of endosomal escape of rNleC is reminiscent of its homolog AIP56, a Zn-metalloprotease short-trip A-B toxin identified in the fish pathogen *Photobacterium damselae piscicida* (*Phdp*) (Pereira et al., 2014). AIP56 qualifies as a short-trip toxin as it escapes already from endosomes in contrast to long-trip toxins, such as Shiga toxin or pertussis toxin undergoing retrograde transport to the endoplasmic reticulum. NleC and the A subunit of AIP56 share high homologies in sequence and structure suggesting a common ancestor. Although NleC and AIP56 both cleave the NF-κB subunit p65, AIP56-dependent p65 cleavage leads to apoptosis, whereas cleavage by NleC results in down-regulation of pro-inflammatory cytokines. How cleavage of p65 by AIP56 might differ compared to NleC and why this ultimately leads to different cellular responses has not been unraveled. However, NleC and AIP56 have never been compared in the same experimental setup, which makes their direct comparison difficult. It remains elusive whether the response of AIP56 in different cells also leads to apoptosis. Although the similarity between the A subunit of AIP56 and NleC has been identified in 2005 (Do Vale et al., 2005), to our knowledge a possible evolutionary link between toxins and effector proteins has never been suggested. Results obtained in this study support an evolutionary connection between the A subunit of the short-trip toxin AIP56 and the T3SS effector NleC and we propose that NleC evolved from a toxin (or a toxin precursor) into a T3SS effector retaining the capacity to enter host cells autonomously.

## Materials and methods

### Tissue culture methods

HeLa cells were regularly cultured in DMEM low glucose (1 g/L) with L-glutamine (Sigma Aldrich, St. Louis, MO, USA), supplemented with 10% fetal bovine serum, 1% non-essential amino acids and penicillin/streptomycin. Cells were cultured at 37°C in a 5% CO_2_ atmosphere. Media was replaced every 2–3 days. For experiments where phosphorylation levels of p65 were determined, cells were serum-starved overnight. Experiments were performed at passage number 24–32. Regular tests demonstrated the cells to be free of Mycoplasma contamination.

### Antibodies and chemicals

The following antibodies were used in the study: NF-κB p65 D14E12 (Cell Signaling Technology, Danvers, MA, USA), phospho-NF-κB p65 (Cell Signaling Technology), FLAG M2 (Sigma-Aldrich, St. Louis, MO, USA), Rab5 (Cell Signaling Technology), Rab6 (Santa Cruz Biotechnology, Dallas, TX, USA), Rab7 (Cell Signaling Technology), CD63 H5C6 (Developmental Studies Hybridoma Bank, Iowa City, IA, USA), beta COP1 (Abcam, Cambridge, United Kingdom), GM130 (Cell Signaling Technology), alpha tubulin DM1A (Sigma-Aldrich), GAPDH FL335 (Santa Cruz), HRP-labeled goat anti rabbit (Dianova, Hamburg, Germany), HRP-labeled goat anti mouse (Dianova), Cy3-labeled goat anti rabbit (Dianova) Cy3-labeled goat anti mouse (Dianova).

The following chemicals were used in this study: Amiloride (Sigma-Aldrich), Bafilomycin A1 (invivoGen, San Diego, CA, USA), BAY 11-7085 (Calbiochem, San Diego, CA, USA), 2′7-bis(2-carboxyethyl)-5(6)-carboxy-fluoresceinacetoxy-methylester (Sigma-Aldrich), 6,P-toluidinyl-naphtalene-2-sulphonate (TNS) (SigmaAldrich), 5-(and 6-) carboxynaphthofluorescein succinimidyl ester, mixed isomers (Thermo Fisher Scientific, Waltham, MA, USA), Cytochalasin D (Sigma-Aldrich), DAPI (Sigma-Aldrich), DAKO Mounting Medium (DAKO, Glostrup, Denmark), Dynasore (Enzo Life Sciences, Farmingdale, NY, USA), Draq5 (BioStatus, Shepshed, United Kingdom), Filipin (Sigma-Aldrich), IL1β (Sigma-Aldrich), Lysotracker red DND99 (Thermo-Fisher Scientific), Methyl-β-cyclodextrin (Sigma-Aldrich), N-Lauroylsarcosine sodium salt (Sigma-Aldrich), Nocodazole (Sigma-Aldrich), Propidium iodide (Sigma-Aldrich), Trypan blue (Sigma-Aldrich).

### *In silico* predictions of PTDs (CPP)

The prediction of the potential (PTDs) was performed by Ü. Langel (CePeP, Sweden) using the prediction tool MPEx (Snider et al., 2009), which is based on the Wimley-White hydrophobicity scale (White and Wimley, 1999).

### Cloning and protein production and purification

Plasmids for the overexpression of proteins were constructed using the pET24b(+) 3xFLAG vector (Lubos et al., 2014) in order to generate rNleC or rTat NleC with C-terminal 3xFLAG tags for detection of the protein and 6xHis for purification. To insert the *nlec* gene into the vector, a restriction-free cloning approach was chosen (Chen et al., 2000; van den Ent and Löwe, 2006) using the primer pairs F-NleC 3xFLAG/R-NleC 3xFLAG or F-Tat NleC 3xFLAG/R-NleC 3xFLAG (Table 1) and genomic EPEC E2348/69 DNA. Restriction-free cloning was performed as described in Lubos et al. (2014). Deletion mutants were generated using an inverse PCR approach with the insertion of restriction sites for *Eco*RI using deletion primer pairs (F-NleC Δ183–187/R-NleC Δ183–187 or F-NleC Δ208–257/R-NleC Δ208–257) employing pET24b(+): NleC 3xFLAG as a template. The resulting PCR product was digested with *Eco*RI and *Dpn*I and ligated. In order to generate rPTD1+2 GFP the sequence for ptd1+2 was inserted into pET24b(+) GFP 3xFLAG (Lubos et al., 2014) via inverse PCR with the primer pairs F-PTD1+2GFP and R-PTD1+2GFP generating pET24b(+) PTD1+2 GFP 3xFLAG. The corresponding primer sequences are listed in Table 1. The resulting PCR product was digested with *Dpn*I and ligated. The plasmid pET24b(+) GFP 3xFLAG had been constructed previously (Lubos et al., 2014). Plasmids were transformed into ClearColi® (Lucigen), expressed at 37°C (OD_600_ = 0.7) and induced with 1 mM IPTG for 4 h. Bacterial cultures were harvested by centrifugation. Bacterial pellets were resuspended in 50 mM Tris HCl, pH 8.0, 500 mM NaCl, 10 mM Imidazole, 10% Glycerol, and 0.1% Triton X-100. Bacterial lysis was performed by sonication. The crude extract was incubated with 2% N-Lauroylsarcosine for 1 h prior to centrifugation at 7,200 × g for 15 min. The soluble fraction was added to Protino® Ni-NTA Agarose (Macherey-Nagel) for 1 h at 4°C with rotation. The resin was washed twice with 50 mM Tris HCl, pH 8.0, 500 mM NaCl, 20 mM Imidazole, 10% Glycerol, and 0.5% Triton X-100 and once in 50 mM Tris HCl, pH 8.0, 500 mM NaCl, 20 mM Imidazole, 10% Glycerol, and 0.1% Triton X-100. Proteins were eluted from the Ni-NTA agarose matrix with 50 mM Tris HCl, pH 8.0, 500 mM NaCl, 60 mM Imidazole, and 10% Glycerol, dialyzed twice against phosphate-buffered saline (PBS) and concentrated using Centricon centrifugal filters (Milipore). The protein concentration was determined by a BCA assay (Thermo Scientific). Proteins were stored at 4°C in PBS until use.

**Table 1:** **Primer sequences**.

**Oligonucleotides**	**Sequence**
R-NleC 3xFLAG	CTT ATC GTC GTC ATC CTT GTA ATC GGA TCC TCG CTG ATT GTG TTT GTC CAC ATC CCC AAA
F-TatNleC 3xFLAG	GGC CGT AAG AAA CGT CGC CAG CGT CGC CGT AAA ATT CCC TCA TTA CAG TCC AAC TTC AAC
F-NleC Δ183-187 3xFLAG	CCG GAA TTC CGG CAT GTT ACT GGA TCT AGC GAT
R-NleC Δ183-187 3xFLAG	CCG GAA TTC CGG AAT CAG TCC TTC CTG CCA CGA
F-NleC Δ208-257 3xFLAG	CCG GAA TTC CGG TTC TTC GAA AGG CTG GGT ACG
R-NleC Δ208-257 3xFLAG	CCG GAA TTC CGG AAT CTC GGT GGG TCC TAA CTC
F-PTD1+2 GFP 3xFLAG	CTT TAA GAA GGA GAT ATA CAT ATG GCT AGC CTC GCA CGT CGT GTC GCT CAA GAA CTG GGA
R-PTD1+2 GFP 3xFLAG	GAA AAG TTC TTC TCC GCT TAC ACT AGT GCC AGC CCT CTC ATT CTC TTC ATG CCT CAT GGC

### Cell-based rNleC activity assays *in vitro*

To assess the catalytic activity of rNleC and of the different fusion/deletion versions of NleC, 2.2 × 10^6^ cells were seeded into a 10 cm dish (one 10 cm dish per sample). The following day, HeLa cells were lysed with 350 μl cold radioimmunoprecipitation assay buffer (RIPA buffer: 25 mM Tris-HCl pH 8.0, 137 mM NaCl, 0.1% /w/v) SDS, 0.5% (w/v) Na-deoxycholate, 10% (v/v) Glycerol, 1% (v/v) Nonident P40) in the absence of protease inhibitors and gentle agitation for 30 min at 4°C. Lysates were pooled and cell debris was removed by centrifugation at 10,000 × g for 10 min at 4°C. 350 μl of the supernatants were incubated with 250 μg protein for 30 min at 4°C. This equals the maximum amount of protein that was added to cell supernatants to test whether the applied amount of protein was sufficient to cleave p65. 25 μg/ml equals 576 nM rNleC, 560 nM rTatNleC, 581 nM rNleC Δ183–187, 659 nM rNleC Δ208–257, 827 nM rGFP, or 662 nM rPTD1+2 GFP. The reaction was stopped by the addition of 4x Laemmli buffer and subsequently the cellular proteins were denatured by incubation for 10 min at 95°C. The enzymatic activity was confirmed by Western blotting using an α-NF-κB p65 antibody (1:1,000 dilution in 0.5% skim milk, 0.1% Tween 20 in PBS) for detection of p65 and its cleavage product. To examine the ability of rNleC to cleave p65 in intact cells, cells were seeded in 10 cm dishes, grown to 80% confluence and serum starved overnight. Cells were incubated for up to 4 h in a volume of 5 ml with recombinant protein with the indicated concentration and stimulated with 10 ng/ml IL1β for 20 min unless indicated otherwise. In order to block activation of the NF-κB signaling cascade, cells were pre-incubated with the inhibitor BAY 11-7085 (10 μM/1 h). For inhibiting endosome acidification 10 nM Bafilomycin A1 was added to the cells 2 h prior to protein incubation and subsequent IL1β stimulation. Cells were lysed in RIPA buffer for 30 min at 4°C and—for removal of cell debris—the lysates were centrifuged with 16,000 × g at 4°C for 10 min. 4 × Laemmli buffer was added and the samples were denatured further for 10 min at 95°C. For Western blotting, proteins were transferred onto nitrocellulose membranes using a semi-dry transfer chamber (Transblot SD Semi-dry transfer cell, BioRad Laboratories) for 1 h at 15 V. Unspecific binding sites were blocked with 5% skim milk, 0.1% Tween 20 in PBS for 1 h at RT. Primary antibodies were incubated at RT for 1 h or overnight at 4°C with α-NF-κB p65 (1:1,000 dilution in 0.5% skim milk, 0.1% Tween 20 in PBS), α-phospho-NF-κB p65 (1:1,000 dilution in 0.5% skim milk, 0.1% Tween 20 in PBS) antibodies. The recombinant proteins were detected in lysates with α-FLAG (1:1,000 dilution in 0.5% skim milk, 0.1% Tween 20 in PBS) antibody. As loading control, we used the reaction with α-tubulin (1:1,000 dilution in 0.5% skim milk, 0.1% Tween 20 in PBS) or α-GAPDH (1:1,000 dilution in 0.5% skim milk, 0.1% Tween 20 in PBS) antibodies. Enzymatic activity of rNleC and its derivatives was monitored by the formation of a p65 double band. For detection of primary antibodies, peroxidase labeled goat α-mouse, or goat α-rabbit secondary antibodies were incubated with the nitrocellulose membranes for 1 h at RT in a 1:10,000 dilution in 0.5% skim milk, 0.1% Tween 20 in PBS. Blots were washed in between incubation times three times 0.1% Tween in PBS. For detection, Pierce™ (ECL) Western Blotting Substrate (Thermo Fisher Scientific) was used as a substrate. The detection of emitted light was performed using a Lumi-Imager F1 (Boehringer Mannheim) with the software LumiAnalyst 3.1 (Boehringer Mannheim) (**Figure 2**) or Odessey Fc (Li-Cor Biosciences) with the software ImageStudio™ Lite (Li-Cor Biosciences) (**Figures 3B**, **5B**, **8B** and Figure S6A). Densitometric analysis was performed with the corresponding software programs. For evaluation, band intensities of cleaved p65 (lower p65 band) was divided by band intensities of total p65 (the sum of uncleaved (upper) and cleaved (lower) p65 band).

### Flow cytometry

For studying uptake kinetics of the different recombinant proteins continuous time-lapse quenched uptake assays were performed as described previously (Lubos et al., 2014). Briefly, trypsinized HeLa cells were incubated with 25 μg/ml fluorescein isothiocyanate (FITC)-labeled proteins or 576 nM FITC-labeled peptides. Proteins were labeled using Fluorescein Isothiocyanate (FITC) the FluoReporter® FITC Protein labeling Kit (Thermo Fisher Scientific, Waltham, MA, USA) and labeling was conducted following the manufacturer's instructions. FITC-labeled peptides were synthetized and purchased from GenScript (Piscataway, NJ, USA). At the indicated time points samples were taken, quenched with 0.2% Trypan Blue (final concentration) and subjected to FACS analysis using a FACScan flow cytometer (BD BioScience). To measure a potential influence of the recombinant proteins on the integrity of cellular membranes, cells were incubated with 1 μg/ml propidium iodide (PI) in parallel. In order to block different endocytic uptake routes, cells were pre-incubated for 1 h with the appropriate inhibitor (30 mM dynasore to block dynamin—and clathrin-mediated endocytosis, 200 μM cytochalasin D to block F-actin elongation, 16.5 mM nocodazole to block microtubule polymerization, 19 mM amiloride to inhibit macropinocytosis, and 3.8 mM filipin or 50 mM methyl-β-cyclodextrin to block lipid raft mediated endocytosis). Cells were incubated with 25 μg/ml FITC-labeled recombinant proteins for 4 h at 37°C, washed with PBS, trypsinized, quenched with a final concentration of 0.2% Trypan Blue and immediately measured using the FACScan flow cytometer (BD BioScience). To measure the impact of protein on the acidification of endosomes, 50 μg/ml protein were incubated with HeLa cells. 3 h prior to the end of an experiment, 400 nM lysotracker DND99 was added to the cells to stain acidified endosomes. Cells were washed, trypsinized and subjected to FACS analysis using a FACScan flow cytometer (BD BioScience). To only measure protein residing in a neutral environment within the cell, endpoint assays with 5-(and 6-)carboxynaphthofluorescein (NF)-labeled proteins were conducted. Proteins were labeled using the dye 5-(and-6)-carboxynaphthofluorescein succinimidyl ester mixed isomers (Thermo Fisher Scientific, Waltham, MA, USA) and labeling was performed according to the manufacturer's instructions. For the uptake assays with NF-labeled proteins, HeLa cells were grown to 80% confluence in a 6-well plate format and incubated with 25 μg/ml NF-labeled protein for the indicated times. To remove putative extracellular signals the cells were washed with PBS and quenched with 0.2% glycine in PBS for 5 min. Cells were trypsinized and fluorescence intensities were measured using FACScan flow cytometer (BD BioScience). To exclude cross-signals arising from proteins residing in neutral early endosomes, pulse-chase assays were performed with NF-labeled proteins. Cells at 80% confluence were pre-chilled on ice for 30 min, followed by incubation with 50 μg/ml NF-labeled protein for additional 30 min on ice and then the cells were transferred to 37°C for 1 h. Cells were washed with PBS and fresh media was added for the indicated times. To quench signals from the outside of the cell, cells were incubated with 0.2% glycine in PBS for 5 min and trypsinized. Cells were directly measured using FACScan flow cytometer (BD BioScience). In each FACS experiment the fluorescence intensity (GeoMean) of 10,000 cells was measured in triplicates. Each experiment was conducted at least three times.

### Confocal laser scanning microscopy

HeLa cells were seeded on cover slips and grown over night. Labeled proteins or peptides were added to cells in the indicated concentrations and at the given time points. To stain acidified lysosomes, 400 nM Lysotracker DND99 was incubated 3 h prior to the end of an experiment. Cells were washed twice for 20 min with 0.05% Tween, fixed with 4% paraformaldehyde (PFA) for 20 min at RT, quenched with 0.2% glycine in PBS and permeabilized with 0.2% Triton X 100. For co-localization studies, the specimen were blocked in 5% goat serum in PBS and incubated with the appropriate antibodies for 1 h at RT. In order to demonstrate co-localization with different markers of the endosomal-lysosomal pathway, specimen were visualized with primary antibodies raised against Rab5 (1:50) as a marker for early endosomes, Rab7 (1:25) as a marker for late endosomes and CD63 (1:600) as a marker for late endosomes/early lysosomes. In order to investigate co-localization with markers of the retrograde trafficking route, specimen were incubated with primary antibodies raised against β-COP1 (1:100) as a marker for trafficking endosomes, Rab6 (1:100) to mark the trans-Golgi/ER network and exocytic vesicles or GM130 (1:25) to stain for the Golgi apparatus. For detection the appropriate α-mouse or α-rabbit Cy3-labeled antibodies were employed. Where indicated, filamentous actin was stained with Phalloidin-TRITC (Sigma) for 30 min at RT in a 1:500 dilution. The nucleus was stained with DAPI (1:500) (Sigma) or Draq5 (1:500) (Biostatus) for 30 min at RT. Cells were washed 3 times with PBS after each step. Before mounting in DAKO mounting medium, cover slips were washed once in ddH_2_O. Cells were visualized using a Zeiss LSM 510, Zeiss LSM 780, or Zeiss LSM 800 confocal laser-scanning microscope. Quantification of co-localization was performed with the free available BioImageXD software (Kankaanpää et al., 2012). Briefly, LSM files were uploaded to BioImage XD to analyze a pixel-based co-localization between two channels of interest. Thresholds were adjusted automatically and statistics were calculated to obtain reliable quantitative results.

### Lactate dehydrogenase (LDH)-based cytotoxicity assay

To measure the release of LDH in the culture medium, 1 × 10^4^ HeLa cells per well were seeded in a 96 well plate. The next day, 50 μg/ml recombinant protein was incubated with HeLa cells for 1, 4, and 24 h in a volume of 100 μl. The quantification of LDH was performed using the CytoTox 96® Non-Radioactive Cytotoxicity Assay kit (Promega GmbH, Mannheim, Germany) which is based on a colorimetric two-step enzymatic reaction. The assay was performed following the manufacturer's instructions.

### Measurement of conformational changes in proteins using TNS fluorescence

Conformational changes of recombinant proteins *in vitro* were measured using 6,P-toluidinyl-naphthalene-2-sulfonate (TNS) (Sigma-Aldrich) (Albani, 2009). 1.5 μM recombinant protein was incubated in buffers ranging from pH 7.5 to pH 4.0 (150 mM NaCl, 100 mM ammonium acetate adjusted to pH 4.0, 4.5, 5.0, and 5.5, 150 mM NaCl, 100 mM MOPS adjusted to pH 6.0 and 6.5 or 150 mM NaCl, 100 mM HEPES adjusted to pH 7.0 and 7.5) together with 150 μM TNS for 15 min at RT in non-autofluorescent 96 well plates (black, 655079, Greiner) TNS fluorescence was measured using the photospectrometer SpectraMax MS (Molecular Devices GmbH) (excitation wavelength: 270 nm, emission spectra: 400–500 nm). The background fluorescence (buffer + TNS) was subtracted from the signal. For easier comparison of the capacity to alter their conformations between different recombinant proteins, the fold change between pH 7.5 and pH 4.0 was calculated.

### Database screening for homologous proteins

We used blastp (including or excluding *E. coli*) to reconstruct the affiliations of NleC to AIP56 and APSE-2 ORF D. A subsequent blastp using all three queries revealed the complex pattern of related sequences shown in **Figure 9A**.

### Alignment of protein sequences

The multiple sequence alignment tool Clustal Omega (http://www.ebi.ac.uk/Tools/msa/clustalo/) was used for pre-aligning protein sequences under default settings. This alignment was used for a manual curation and removal of non-overlapping sequence regions in Se-Al Sequence Alignment Editor v2.0a11 (http://tree.bio.ed.ac.uk/software/seal/). **Figure 9B** shows a schematic representation of the aligned sequence regions. The final alignment is presented in fasta format as Figure S11. For AA identity or similarity calculations, we used the NCBI blastp suite for aligning two sequences.

### SplitsTree

The SplitsTree4 software (version 4.10) was used for computing an unrooted phylogenetic network. Settings were *Uncorrected_P* distance, *NeighborNet*, and bootstrap with 10,000 replicates.

## Results

### Identification of motifs potentially promoting uptake and/or intracellular trafficking

**Figure 1:**
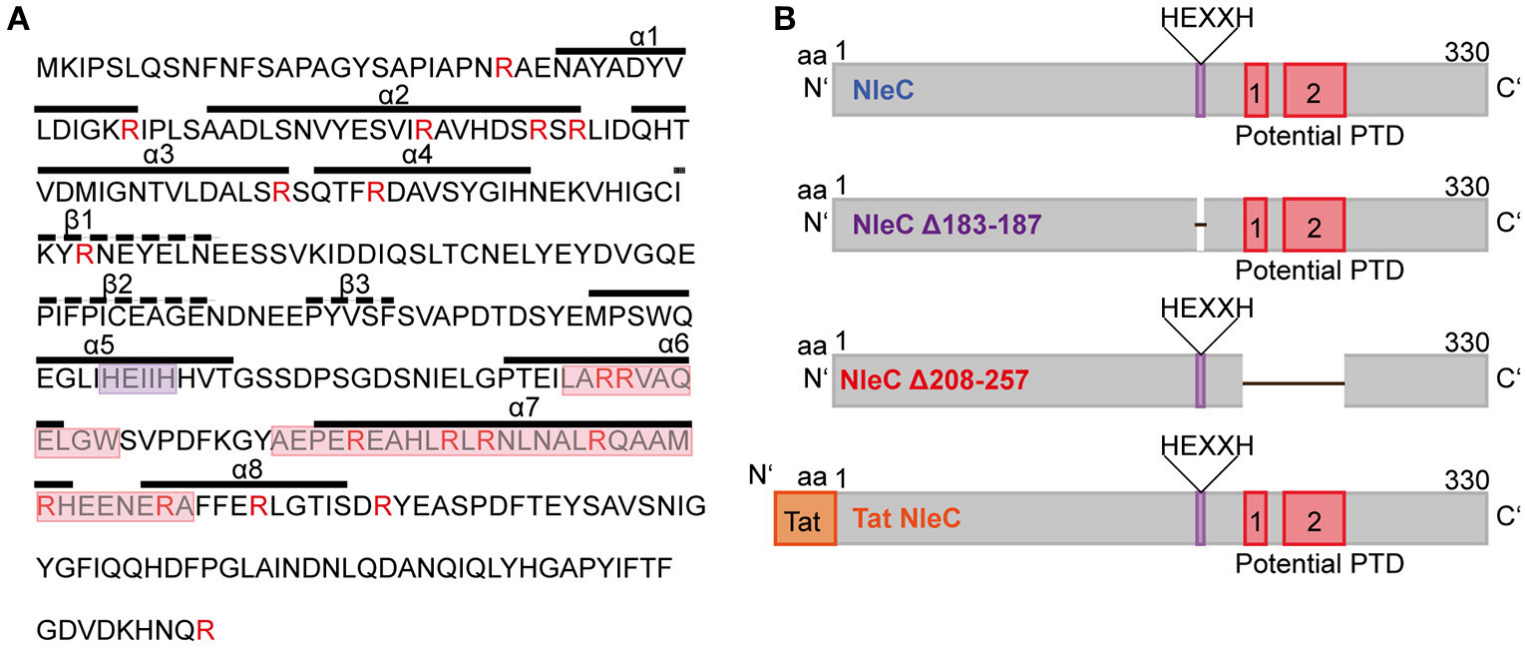
**NleC is predicted to harbor two distinct protein transduction domains. (A)** Primary and secondary structures of NleC. Potential protein transduction domains (red box) and the catalytic center (purple box) are highlighted. Arginine residues are marked in red. A-helices and β-sheets are visualized with straight and dotted lines, respectively. **(B)** Schematic view of NleC and NleC variants showing the wild-type protein (NleC), a catalytically inactive mutant lacking the HEXXH motif (NleC Δ183-187), and a mutant lacking the potential PTDs (NleC Δ208-257). Further the N-terminal position of the Tat CPP is indicated in the rTat NleC fusion protein.

In our previous work, we found the effector proteins YopM from *Y. enterocolitica* and SspH1 from *S*. *enterica* serovar Typhimurium to have cell-penetrating abilities (Rüter et al., 2010; Lubos et al., 2014). Hence, we investigated whether also other effector proteins might share this ability to overcome membranes of targeted host cells. Therefore, we first analyzed known effector proteins of EPEC *in silico* for the presence of putative protein transduction domains (PTDs). Here, we identified NleC, a zinc-dependent metalloprotease, which cleaves p65 of the NF-κB pathway and thus contributes to the abrogation of the host's innate immune response. The *in silico* analysis indicated that NleC might potentially harbor two PTDs near the C-terminus. These regions form α-helical structures and exhibit a high arginine content, which would favor attachment to and uptake across eukaryotic membranes (Figure 1A and Figure S1A; Tang et al., 2013). For further analysis, we generated additional versions of NleC (Figure 1B). For rNleC Δ183–187 the HEXXH catalytic motif as part of the catalytic domain has been deleted. This version should be taken up by host cells, but should be catalytically inactive and unable to cleave p65. rNleC Δ208–257 lacks the *in silico*-predicted PTDs and should thus be unable to get across eukaryotic membranes, however, should be catalytically active. Our initial strategy to control catalytic activity included a point mutation of histidine 187 to tyrosine, which was shown in a previous publication to abolish the catalytic activity of NleC (Yen et al., 2010). The validity of the point mutation had been confirmed by sequencing. However, when we investigated the activity of this mutant in HeLa cell lysates, we observed quite some residual catalytic activity (Figure S1B). Therefore, we decided to delete the whole HEXXH signature. rTat NleC is a fusion protein of NleC with the well-described CPP Tat (“transactivator of transcription”; Frankel and Pabo, 1988) at the N-terminus. This protein served as a control to compare its ability to enter cells with that of the wt rNleC and of the two deletion mutants (Figure 1B).

### For p65 cleavage rNleC requires an activated NF-κB signaling cascade

**Figure 2:**
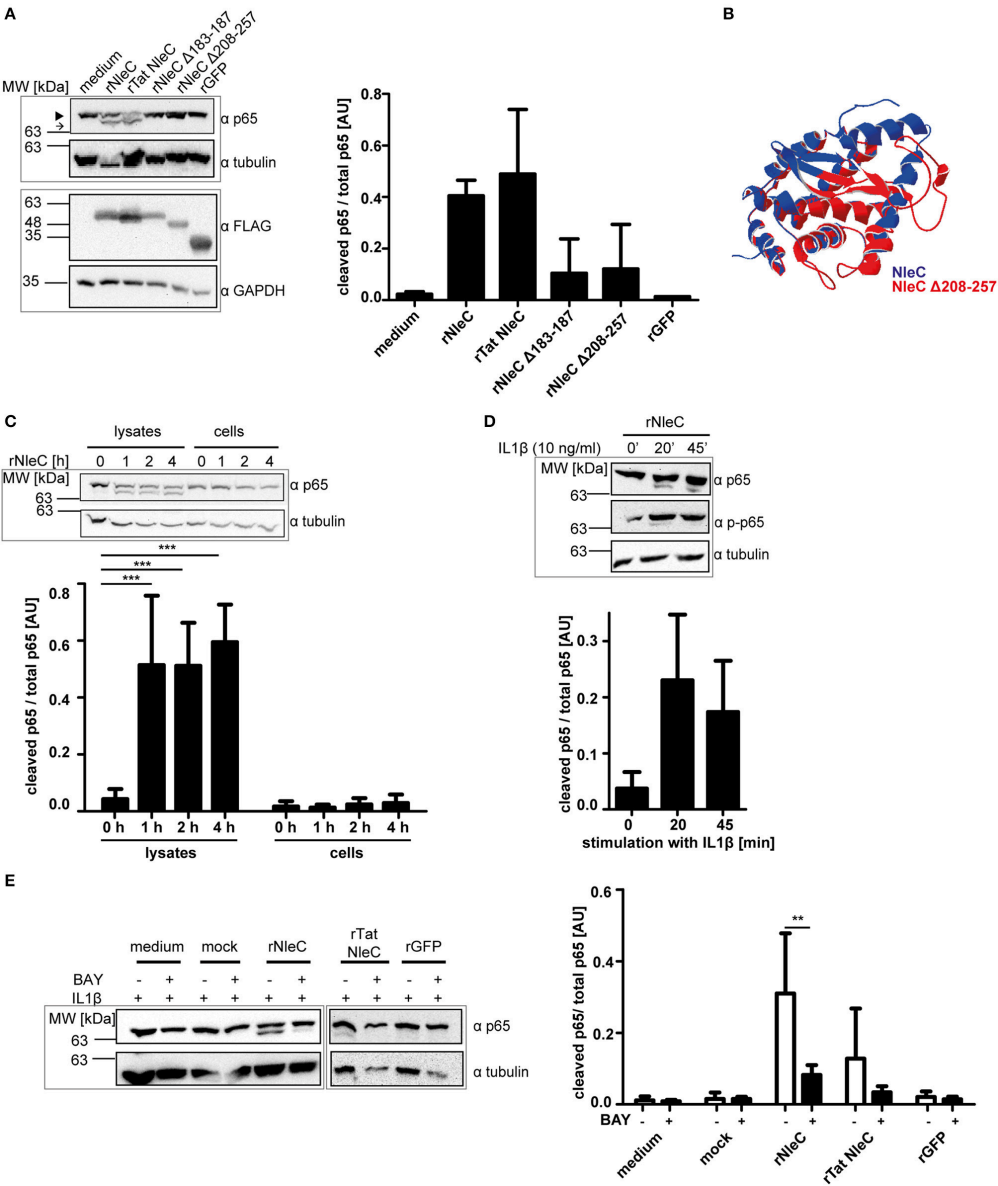
**rNleC is catalytically active but requires IL1β-stimulated cells for p65 cleavage. (A)** 250 μg of rNleC variants were incubated for 30 min with HeLa cell lysates at 4°C and analyzed for p65-cleaving events by Western blotting. The closed arrowhead marks the full-length proteins, the arrow highlights the cleaved proteins. The bar graph depicts the densitometric evaluation of immunoblots of at least three independent experiments (mean ± SD) with the levels of significance (one-way ANOVA, followed by Bonferroni's multiple comparisons test) indicated (see below). FLAG-tagged rNleC, rNleC variants and rGFP were detected with an α-FLAG antibody. **(B)** Overlay of predicted 3D structures of NleC (blue) and NleC Δ208-257 (red) generated by Swiss-PdbViewer 4.1.0. **(C)** rNleC was incubated for the indicated time intervals with HeLa cell lysates (125 μg rNleC at 4°C) or whole cells (25 μg/ml rNleC at 37°C). Immunoblots for p65 were done to detect rNleC-dependent cleavage events. The bar graph shows the densitometric evaluation of immunoblots of at least three independent experiments (mean ± SD) with the levels of significance (two-way ANOVA, followed by Bonferroni's multiple comparison test) indicated (see below). **(D)** HeLa cells were incubated with 25 μg/ml rNleC for 1 h at 37°C and stimulated with 10 ng/ml IL1β for the indicated times. Cleaving events and stimulation were visualized with immunoblots for p65 and phosphorylated p65, respectively. Bar graph shows densitometric evaluation of immunoblots of at least three independent experiments (mean ± SD) with the levels of significance (one-way ANOVA, followed by Bonferroni's multiple comparison test) indicated (see below). **(E)** HeLa cells were pre-incubated with BAY 11-7085 inhibitor (10 μM) for 1 h, followed by incubation with rNleC (50 μg/ml) for 1 h at 37°C. Cells were stimulated with 10 ng/ml IL1β for 20 min at 37°C prior to lysis in radioimmunoprecipitation assay (RIPA) buffer. Cleavage events of p65 were analyzed by immunoblotting with α-p65 antibodies. Tubulin was used as loading control. The bar graph shows the densitometric evaluation of immunoblots of at least three independent experiments (mean ± SD) with the levels of significance (two-way ANOVA, followed by Bonferroni's multiple comparison test) indicated. ^**^p ≤ 0.01 and ^***^p ≤ 0.001. Gray boxes indicate bands from the same gel.

First, we tested the catalytic functions of rNleC and rNleC deletion mutants *in vitro*. In Figure 2A rNleC and different NleC variants were incubated with HeLa cell lysates. Only rNleC and rTat NleC proteolytically cleaved the NF-κB subunit p65, whereas the deletion mutants were unable to cleave p65. NleC Δ208–257 harbors the HEXXH motif that had been identified as part of the active Zn-binding site and hence serves as a signature for Zn-metalloproteases (Silva et al., 2010) and should in theory be able to cleave p65. However, a comparison of the predictions of the 3D structures for NleC and NleC Δ208–257 suggested extended conformational changes close to the catalytic center in the deletion mutant, which might explain its inability to cleave p65 (Figure 2B). Furthermore, the amino acid Y227, which is deleted in NleC Δ208–257, coordinates the zinc ion and its deletion might result in loss of protease activity (Li et al., 2014; Turco and Sousa, 2014). The functional rNleC protein was tested for catalytic activity in intact HeLa cells. However, rNleC was unable to cleave p65 even after 4 h of incubation (Figure 2C), although the protein was shown to be functional in cell lysates (Figures 2A,C). In non-stimulated cells, NF-κB is retained in the cytosol by the inhibitor κB (IκB), which is phosphorylated and degraded upon stimulation. During EPEC infection the NF-κB signaling cascade is activated due to the recognition of pathogen-associated molecular patterns (PAMPS) and IκB is degraded (Johannessen et al., 2013). In this scenario, T3SS-injected NleC cleaves the p65 subunit of NF-κB, potentially facilitated by the activation of the NF-κB pathway during infection and the ensuing lack of competition with IκB for the p65 substrate. To test this assumption, cells were activated by incubation with IL1β (10 ng/ml), which induces NF-κB signaling (Figure 2D). Indeed, after 20 and 45 min of activation, we could observe cleavage of p65 as indicated by the appearance of the p65 double band in Western blotting. This clearly showed that the NF-κB pathway needed to be activated for efficient cleavage of p65 by rNleC and implies a preference of NleC to free p65 rather than to IκB bound p65. To exclude an impact of IL1β on the integrity of the cells, cells were pretreated with 50 μg/ml rNleC for 30 min together with 1 μg/ml propidium iodide (PI) followed by stimulation with 10 ng/ml IL1β for 3.5 h. The uptake of PI was measured using flow cytometry (Figure S2). No significant differences in PI uptake between IL1β treated and IL1β untreated cells could be observed.

### The NF-κB inhibitor bay 11-7085 abrogates the effect of IL1β activation and prevents p65 cleavage by rNleC

Intact cells need to be activated with IL1β for rNleC to cleave p65 of NF-κB. To demonstrate that this effect was due to the stimulation of the NF-κB pathway, we compared the effects of rNleC and rTat NleC in IL1β-stimulated cells to the effect of rNleC and rTat NleC in cells that had been pre-treated for 1 h with the NF-κB inhibitor BAY 11-7085 which shuts down NF-κB signaling (Figure 2E). Pre-incubation with the inhibitor BAY 11-7085 nearly completely abrogated p65 cleavage by rNleC and rTat NleC in IL1β activated cells compared to BAY 11-7085 untreated cells. In summary, we demonstrated that rNleC only cleaves p65 in stimulated cells with an activated NF-κB pathway since inhibition of NF-κB signaling with the inhibitor BAY 11-7085 also blocks p65 cleavage by rNleC.

### rNleC enters eukaryotic cells autonomously and independently of the T3SS

**Figure 3:**
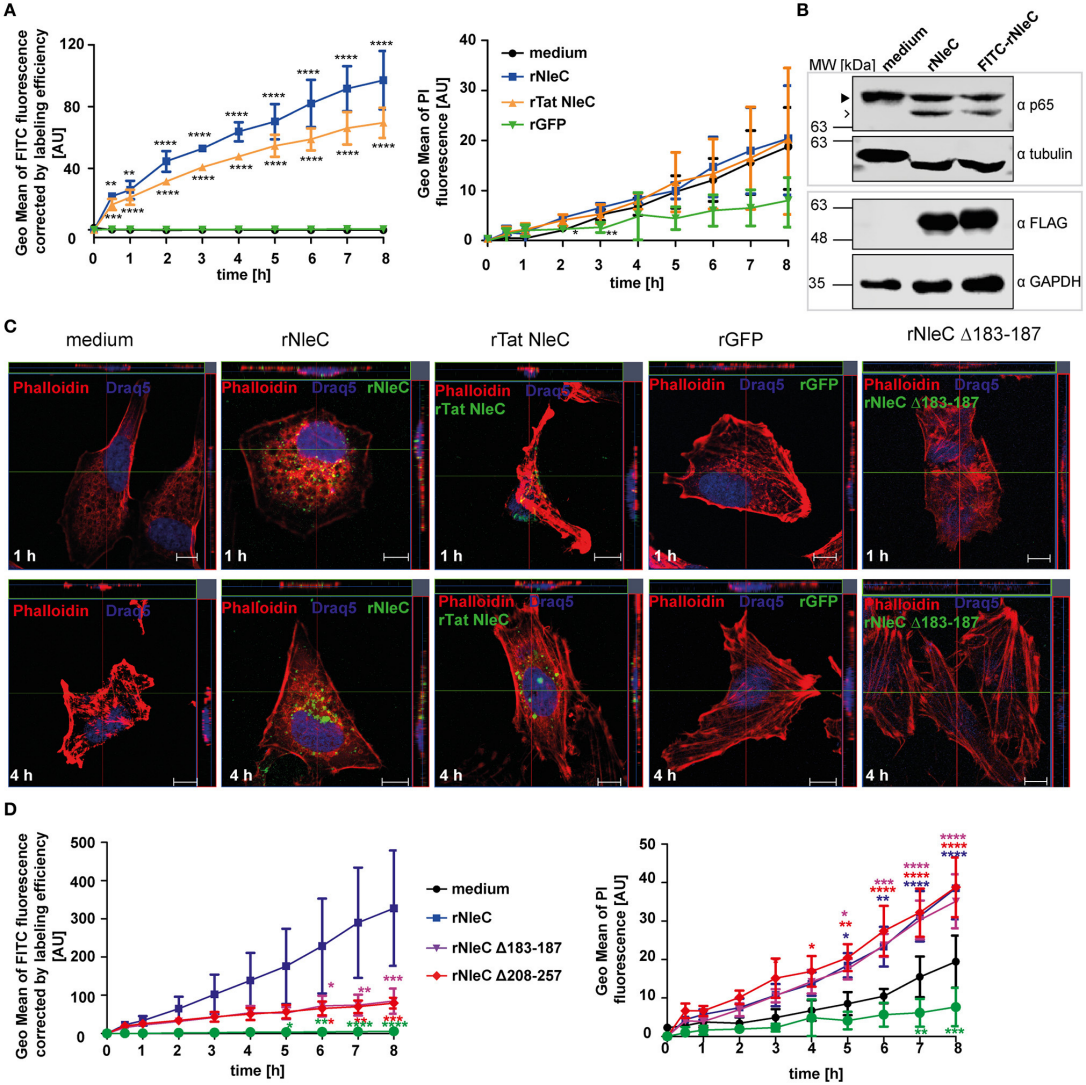
**NleC enters HeLa cells independent of the T3SS. (A)** Trypsinzed HeLa cells were incubated with 25 μg/ml FITC-labeled proteins (rNleC and rTat NleC) or rGFP for the indicated time at 37°C. For quenching extracellular fluorescence Trypan Blue (TB) was added to the cells to a final concentration of 0.2% and fluorescence intensities were detected using flow cytometry. Line graphs are depicted as the mean of at least three independent experiments (mean ± SD) with the levels of significance (two-way ANOVA, followed by Bonferroni's multiple comparison test) compared to rGFP indicated (see below). To exclude cytotoxicity of the recombinant proteins 1 μg/ml PI was added to cells at the beginning of an experiment and its uptake, indicating cell death, was measured (right). Line graphs are depicted as the mean of at least three independent experiments (mean ± SD) with the levels of significance (two-way ANOVA, followed by Bonferroni's multiple comparison test) compared to medium control indicated (see below). **(B)** rNleC and FITC-rNleC were incubated for 30 min with HeLa cell lysates at 4°C and analyzed for p65-cleaving events by Western blotting. The closed arrowhead marks the full-length proteins, the arrow highlights the cleaved proteins. Cleavage of p65 was analyzed by Western blotting using α-p65 antibodies. FLAG-tagged rNleC, was detected with an α-FLAG antibody. Tubulin and GAPDH were used as loading controls. **(C)** Fluorescence microscopy of HeLa cells incubated with 50 μg/ml rNleC-FITC, rTat NleC-FITC (green), or rGFP for 1 h or 4 h at 37°C. PFA fixed cells were stained with Phalloidin TRITC (red) and Draq5 (blue). Fluorescence images were generated using a Zeiss LSM 510 microscope. Z-stacks of single cells were taken. Scale bar represents 10 μm. **(D)** The uptake of 25 μg/ml FITC-labeled proteins (rNleC, rNleC Δ183–187, rNleC Δ208–257) (Figure 3C left) with extracellular fluorescence quenched by Trypan blue and PI uptake (Figure 3C right) was monitored in trypsinized HeLa cells at 37°C. Line graphs are depicted as the mean of at least three independent experiments (mean ± SD) with the levels of significance (two-way ANOVA, followed by Bonferroni's multiple comparison test) compared to rGFP (left graph) or medium control (right graph) indicated (see below). ^*^p ≤ 0.05, ^**^p ≤ 0.01, ^***^p ≤ 0.001, and ^****^p ≤ 0.0001

Previously, Rüter et al. had shown that the T3SS-effector YopM of *Yersinia enterocolitica* as a recombinant expressed and purified protein autonomously entered eukaryotic cells independent of the T3SS thereby revealing properties of a cell-penetrating effector protein (CPE) (Rüter et al., 2010). Using an algorithm developed by Snider et al. we identified several other putative effector proteins of Gram-negative bacteria including SspH1 from *Salmonella enterica* serovar Typhimurium that potentially carry a PTD (Snider et al., 2009; Lubos et al., 2014). In this study, we found that rNleC reached and cleaved its intracellular target p65 when rNleC was added to intact, IL1β-stimulated cells. To further describe the kinetics of rNleC-uptake, FITC-labeled rNleC was added to trypsinized HeLa cells and the uptake of the protein was monitored using a FACS-based time-lapse quenched uptake assay as described by Lubos et al. (2014) (Figure 3A). rNleC showed a linear uptake for up to 8 h which was even more pronounced than internalization of rTat NleC, a fusion protein of rNleC with an established CPP (Hauber et al., 1987). rGFP was not internalized and in this context served as negative control (Figure 3A, left). To exclude that rNleC uptake might be due to cytotoxic effects impairing membrane integrity, the uptake of PI was measured in parallel. Although uptake of PI increased over time, there was no significant difference between cells treated with the various (rNleC) proteins or the medium control indicating that the uptake of PI was not mediated by cytotoxic effects (Figure 3A, right). Importantly, the fluorescent-labeled rNleC retained its catalytic activity as FITC-NleC could proteolytically cleave the NF-κB subunit p65 in HeLa cell lysates (Figure 3B). Uptake of these proteins was visualized additionally by fluorescence microscopy (Figure 3C). FITC-labeled proteins localized in dotted structures within cells after 1 and 4 h of incubation, which suggests their localization in vesicles. rGFP could not be detected inside cells and was employed as a negative control. Interestingly, cellular uptake of rNleC was more pronounced than uptake of rTat NleC. To elucidate whether the rNleC Δ208–257 and rNleC Δ183–187 deletion mutants were able to overcome eukaryotic membranes, the uptake of FITC-labeled rNleC deletion mutants was compared to FITC-labeled rNleC (Figure 3D) in a FACS-based continuous-quenched time-lapse uptake assay. Interestingly, not only the deletion construct lacking the predicted PTDs (rNleC Δ208–257), but also the inactive mutant (rNleC Δ183–187) showed only residual uptake when compared to wild type rNleC. However, based on structure predictions using Swiss-PdbViewer4.1.0., deletions of these amino acid sequence segments might have induced conformational alterations preventing efficient uptake. Hence, the uptake data suggest that already small changes in the three-dimensional structure of NleC reduce uptake efficiency to a basal level (Figure 3D, left). To exclude toxic effects induced by the rNleC Δ208–257 and rNleC Δ183–187 deletion constructs, we measured PI uptake in parallel. In this case, we could observe increased PI uptake following incubation with the recombinant proteins than for the medium control (Figure 3D, right). To further confirm that these deletion mutants did not exert a cytotoxic effect we additionally employed LDH release assays to monitor possible membrane damage. No cytotoxic effects could be observed (Figure S3). To exclude that the observed cleavage (Figure 2D) occurred due to increased uptake of rNleC or rTatNleC after stimulation with IL1β, we incubated 25 μg/ml rNleC FITC for 30 min followed by stimulation with 10 ng/ml IL1β for 3.5 h. Cells were washed trypsinized and quenched using TB and uptake of rNleC-FITC was measured using flow cytometry (Figure S4).

### The predicted protein-transduction domains PTD1 and PTD2 are not responsible for the uptake of rNleC

**Figure 4:**
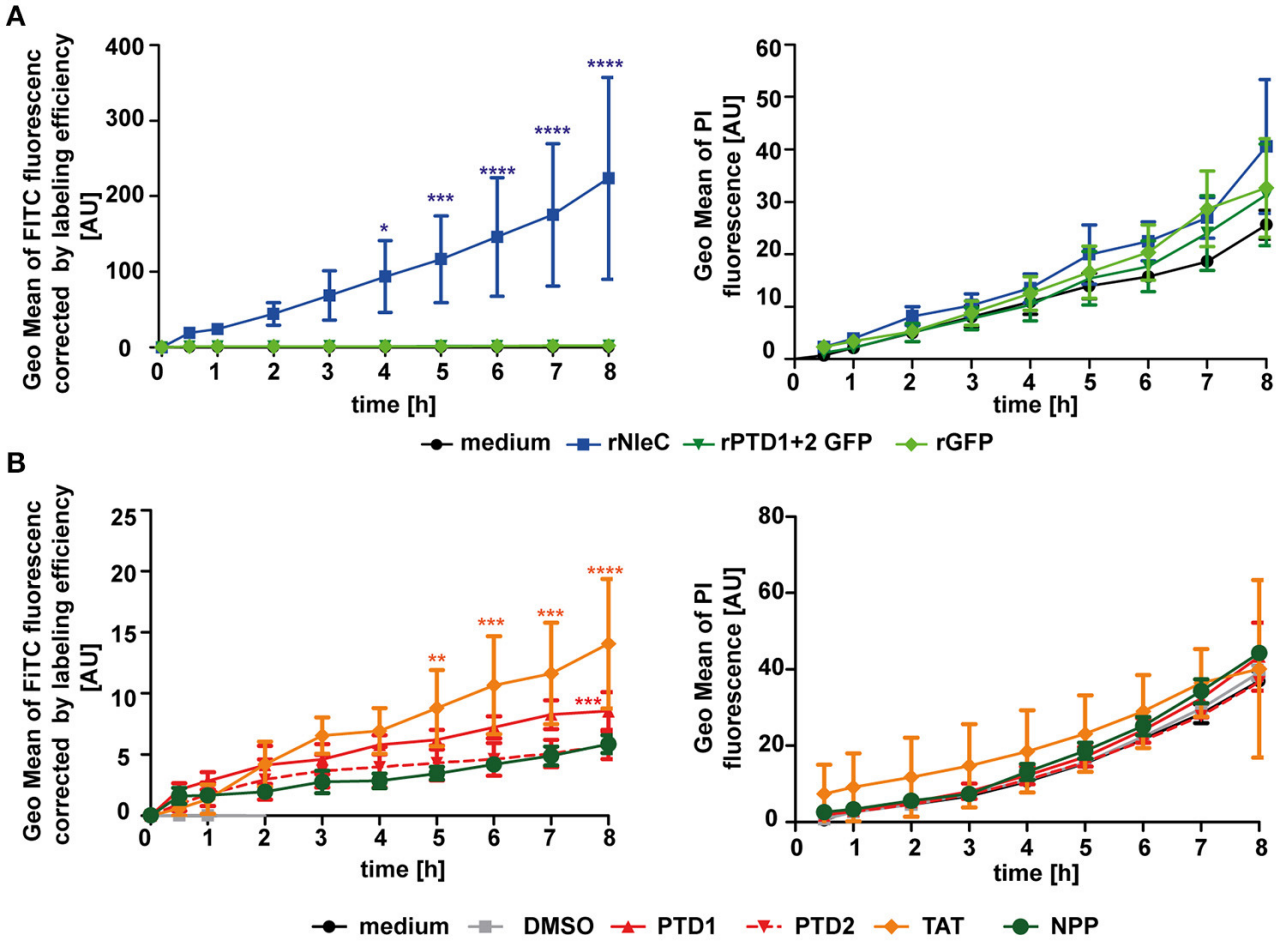
**The predicted PTD1 and PTD2 are not responsible for rNleC uptake. (A)** Trypsinized HeLa cells were incubated with 25 μg/ml FITC-labeled proteins (rNleC or rPTD1 + 2 GFP) and rGFP for the indicated time at 37°C and TB quenched uptake (left) and PI uptake (right) was measured. Line graphs are depicted as the mean of at least three independent experiments (mean ± SD). The levels of significance (two-way ANOVA, followed by Bonferroni's multiple comparison test) compared to rGFP (left graph) or medium control (right graph) are indicated (see below). **(B)** Trypsinized HeLa cells were incubated with 576 nM FITC-labeled peptides (PTD1, PTD2, Tat, or NPP) for the indicated time intervals at 37°C and Trypan Blue-quenched uptake (left) and PI uptake (right) was measured. These experiments were conducted as FACS-based continuous-quenched time-lapse uptake assays. Line graphs are depicted as the mean of at least three independent experiments (mean ± SD) with the levels of significance (two-way ANOVA, followed by Bonferroni's multiple comparison test) compared to NPP (left graph) or medium control (right graph) indicated (see below). ^*^p ≤ 0.05, ^**^p ≤ 0.01, ^***^p ≤ 0.001, and ^****^p ≤ 0.0001.

The *in silico* prediction suggested the presence of two potential PTDs in the C-terminal half of NleC (Figure 1). In accordance with these predictions uptake of the deletion variant rNleC Δ208–257, which lacks both of the predicted domains, was reduced. However, uptake of rNleC Δ183–187 lacking only the HEXXH Zn-binding metalloprotease signature region was also reduced. As CPPs (PTDs) are defined as small peptides, which are able to facilitate also cellular entry of cargo, we investigated whether PTD1 and/or PTD2 could autonomously enter cells and whether they might be able to transport cargo. For this, a FACS-based time-lapse quenched uptake assay was performed with a fusion protein of PTD1+2 and GFP (Figure 4A, left). The uptake of this recombinant protein was compared to FITC-labeled rNleC and to rGFP alone. We did not observe enhanced uptake of rPTD1+2 GFP compared to rGFP, whereas FITC-labeled rNleC was clearly detected inside cells. Cytotoxic effects of rNleC could be excluded since the parallel uptake observed for PI did not differ from the medium control (Figure 4A, right panel). As the uptake efficacy of CPPs might also depend on the nature of the cargos that are fused to the CPP (El-Andaloussi et al., 2007) we investigated the uptake of the FITC-labeled peptides PTD1 and PTD2 without any fused cargo and compared this to the well-described CPP Tat and a non-penetrating peptide (NPP) (Choi et al., 2012) in a FACS-based quenched time-lapse uptake assay as well as by fluorescence microscopy (Figure 4B and Figure S5). We could only observe a low uptake of PTD1 compared to NPP whereas PTD2 uptake did not exceed that of the negative control NPP, suggesting that neither the small increase in uptake for PTD1 nor for PTD2 would explain the remarkable uptake we observed for rNleC. In addition, we could observe a reduction of uptake for the deletion mutant of rNleC Δ183–187 to the same level as rNleC Δ208–257 (Figure 3D). Therefore, in summary, we can exclude that the predicted PTDs mediate the uptake of rNleC and its derivatives. Thus, rNleC cannot be designated as a CPE, as it does not fulfill the two main criteria for CPPs/CPEs: The predicted PTDs cannot enter eukaryotic cells and they are neither necessary nor sufficient to transport cargo into cells. Thus, far, our overall data suggest, that uptake of rNleC depends mostly on the native conformation of NleC and appears not to be mediated by distinct linear segments of amino acids as it is the case for CPEs.

### Endocytic uptake of rNleC proceeds via lipid rafts

Thus far, we have demonstrated efficient uptake of rNleC by eukaryotic cells (Figures 3A,C). However, the mechanism underlying the uptake process has not been addressed. Therefore, we asked whether uptake of the protein might proceed by an endocytic pathway which is followed by many viruses, CPE and bacterial toxins (Sandvig and van Deurs, 1996; Lakadamyali et al., 2004; Rüter et al., 2010).

**Figure 5:**
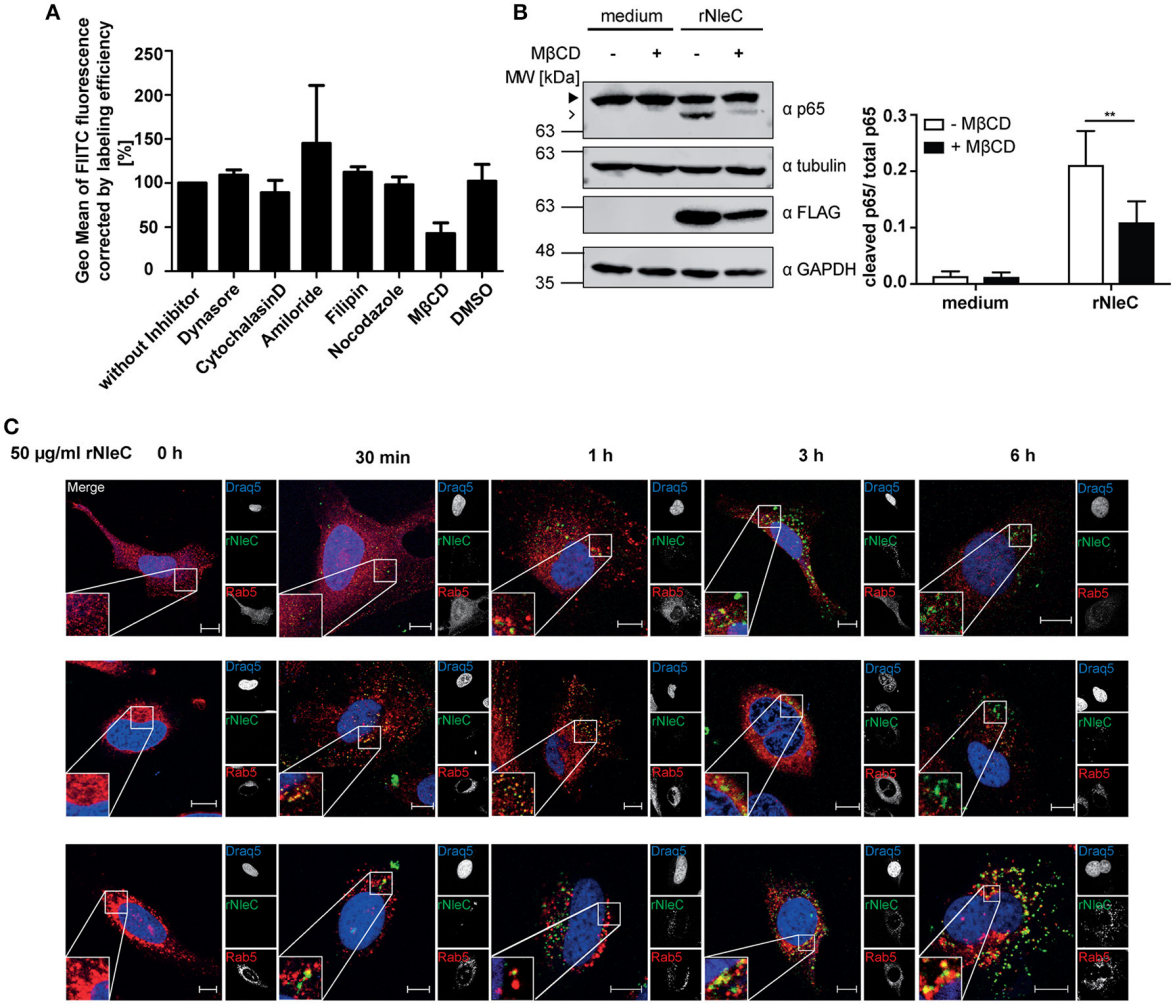
**rNleC enters eukaryotic cells by endocytosis via lipid rafts and is subsequently routed via the endosomal-lysosomal pathway. (A)** Adherent HeLa cells were pre-incubated with the indicated endocytosis inhibitors for 1 h and treated with FITC-labeled rNleC (50 μg/ml) for 4 h at 37°C. Trypsinized cells were quenched with Trypan Blue (final concentration 0.2%) and fluorescence was subsequently measured by flow cytometry. The bar graph shows the results of at least three independent experiments (mean ± SD) with the levels of significance (one-way ANOVA, followed by Bonferroni's multiple comparison test) indicated (see below). **(B)** Adherent HeLa cells were pre-incubated with MβCD (50 mM) for 1 h and treated with rNleC (50 μg/ml) for 4 h at 37°C. Cells were stimulated with 10 ng/ml IL1β for 20 min at 37°C. Cells were lysed in RIPA buffer for 30 min at 4°C. Cleavage of p65 was analyzed by Western blotting using α-p65 antibodies. FLAG-tagged rNleC, was detected with an α-FLAG antibody. Tubulin and GAPDH were used as loading controls. The bar graph shows the results of at least three independent experiments (mean ± SD) with the levels of significance (two-way ANOVA, followed by Bonferroni's multiple comparison test) indicated (see below). gray boxes indicate bands from the same gel. **(C)** Fluorescence microscopy of HeLa cells incubated with 50 μg/ml FITC-labeled rNleC for the indicated time points at 37°C. PFA-fixed cells were stained with Rab5, Rab7 and CD63 and Cy3-labeled secondary antibodies (red) to show co-localization with early endosomes (Rab5) late endosomes (Rab7), and early lysosomes (CD63). Draq5-staining (blue) was used to visualize the nucleus. Fluorescence images were generated using a confocal laser-scanning microscope (Zeiss LSM 510). Z-stacks of single cells were taken. Scale bars represent 10 μm. ^**^p ≤ 0.01.

To identify which endocytic pathway might be important for efficient rNleC uptake, HeLa cells were pre-incubated with different endocytosis inhibitors for 1 h followed by incubation of FITC-labeled rNleC for 4 h. After trypsinization, Trypan Blue-quenched fluorescence was measured using a cytometric approach (Figure 5A). Additionally, it was excluded that the presence of endocytic inhibitors reduces catalytic activity of rNleC (Figure S6A). A reduction in the uptake of more than 50% could be observed only for those cells pre-treated with methyl-β-cyclodextrin (MβCD), an inhibitor of lipid-raft mediated endocytosis. Inhibitors, such as cytochalasin D, which blocks actin-mediated processes, dynasore, which inhibits dynamin—and clathrin-mediated uptake, or nocodazole preventing microtubule—and clathrin-mediated uptake, did not reduce the uptake of rNleC significantly. Also, amiloride, an inhibitor blocking Na^+^-channels and thus micropinocytosis, or filipin, which binds to cholesterol, did not affect uptake of rNleC (Figure 5A). A putative cytotoxicity of the different inhibitors was monitored by measuring uptake of PI (1 μg/ml added together with rNleC) in parallel (Figure S7). Moreover, pre-incubation with MβCD strongly abrogated p65 cleavage by rNleC in IL1β activated cells compared to untreated cells (Figure 5B), indicating that the major uptake route for rNleC is lipid raft-mediated endocytosis.

### rNleC co-localizes with markers of the endosomal-lysosomal pathway

As rNleC very likely enters cells via endocytosis we were interested in the subsequent trafficking of the endocytosed protein. Endosomal contents can be sorted into different endocytic routes, e.g., the endosomal-lysosomal route, which ultimately leads to degradation of the endocytosed material (Hu et al., 2015), retrograde trafficking along the trans-Golgi network and ER (Johannes and Popoff, 2008), or exocytosis which leads to recycling to the outside of the cell (Ward et al., 2005). To investigate the fate of intracellular rNleC, we analyzed a potential co-localization of FITC-labeled rNleC with markers of the endosomal-lysosomal pathway by fluorescence microscopy (Figure 5C). Markers for the endosomal-lysosomal pathway included Rab5 for early endosomes, Rab7 for late endosomes and CD63 for late endosomes/early lysosomes (Metzelaar et al., 1991; Christoforidis et al., 1999; Vanlandingham and Ceresa, 2009). A low co-localization of rNleC with Rab5 was detected after 30 min and 1 h. However, already after 1 h Rab5 co-localization could no longer be observed which is indicative of the well-documented fast shuttling through early endosomes. Between 30 min and 3 h rNleC strongly co-localized with Rab7 and subsequently also with CD63. These results strongly point toward uptake of rNleC via endocytosis with subsequent routing to lysosomes. To address whether rNleC could escape from endosomes or would undergo retrograde trafficking as it is known for example for Shiga toxin or other “long-trip” A-B toxins (Sandvig et al., 2010), we performed co-localization studies with markers for retrograde trafficking, such as Rab6, COP1, and GM130 (Figure S6B) (Nakamura et al., 1995; Grigoriev et al., 2007; Beck et al., 2009). As none of these markers co-localized with rNleC, we excluded retrograde transport as a possible mechanism for endosomal escape of rNleC.

### rNleC co-localizes with lysosomes, but is only partially degraded

**Figure 6:**
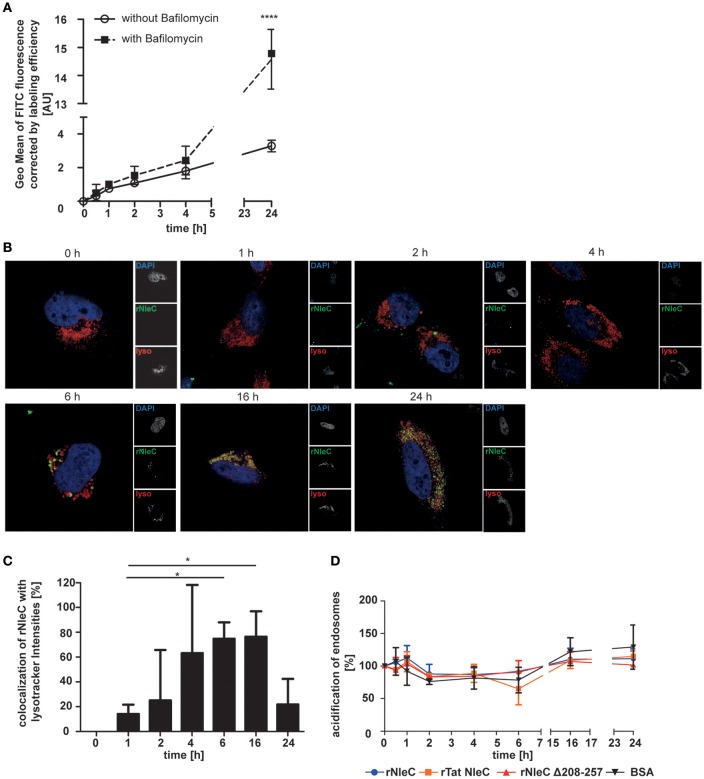
**rNleC is only partially degraded in lysosomes. (A)** FACS-based quenched endpoint uptake assay of 50 μg/ml rNleC FITC with or without pre-incubation with Bafilomycin A1 at 37°C. Cells were washed, trypsinized, and quenched with Trypan Blue prior to analysis using flow cytometry. The data are presented after subtraction of background fluorescence, corrections for labeling efficiency and are depicted as the mean ± SD of three independent experiments. Line graph is depicted as the mean of at least three independent experiments (mean ± SD) with the levels of significance (two-way ANOVA, followed by Bonferroni's multiple comparison test) compared to treatment without Bafilomycin indicated (see below). **(B)** Fluorescence microscopy of HeLa cells which were incubated with 50 μg/ml FITC-labeled rNleC (green) for the indicated times at 37°C. For the last 3 h of an experiment, cells were incubated with 400 nM Lysotracker red DND99 (red). Cells were washed, fixed and the nucleus was stained with DAPI (blue). Fluorescence images were generated using a LSM 800 Zeiss microscope. Scale bars represent 10 μm. **(C)** Quantification of co-localization of rNleC FITC and Lysotracker red DND99 using the software BioImageXD. The bar graph shows the evaluation of at least three microscopic fields (mean ± SD) with the levels of significance (one-way ANOVA, followed by Dunnett's multiple comparison test) indicated (see below). **(D)** Quantification of acidified endosomes labeled with Lysotracker red DND99 upon incubation with 50 μg/ml different recombinant proteins at 37°C. The data are presented after subtraction of background fluorescence and are the mean ± SD of three independent experiments with the levels of significance (two-way ANOVA, followed by Bonferroni's multiple comparison test) compared to BSA indicated (see below). ^*^p ≤ 0.05 and ^****^p ≤ 0.0001.

To investigate whether and to what extend rNleC is degraded by lysosomes we quantified the fluorescence signal of cells treated with Bafilomycin A1, a potent inhibitor of H^+^-ATPases (V-ATPases) which blocks the acidification of lysosomes and thus inhibits lysosomal degradation, and compared it to cells treated with rNleC-FITC without inhibitor (Figure 6A). Our results indicated that partial degradation of the protein took place at 24 h of incubation. As an internal control, we confirmed the functionality of Bafilomycin A1 by quantifying the percentage of acidified lysosomes stained with Lysotracker red DND99 (Figure S8). However, we could still measure an increase in protein uptake after 24 h in Bafilomycin untreated cells, indicating that more protein was taken up after 24 h than degraded. To visualize degradation with lysotracker, we performed co-localization with the pH-sensitive marker Lysotracker red DND99. We confirmed, that rNleC co-localized with acidified lysosomes after 6 h, which could lead to a clearance of the protein from the eukaryotic cell (Figure 6B). Yet, we found rNleC residing in lysosomes for up to 24 h, indicating that, although lysosomes are functional, rNleC is not cleared completely from the cells after 24 h of incubation (Figure 6B). Although relative values compared to Bafilomycin-treated cells seemed to be relatively low, the absolute fluorescence in the cell was indeed significant. Furthermore, a more diffuse signal for rNleC after 16 h and especially after 24 h was observed, indicating that rNleC might have escaped from endosomes and has entered the cytosol. When quantifying the co-localization of rNleC and Lysotracker red DND99, we observed an increase in co-localization up to 16 h, whereas the co-localization decreased after 24 h of rNleC incubation, further indicating a separation of rNleC from lysosomes (Figure 6C). Incubation of cells with rNleC and rNleC variants suggested that these recombinant proteins had no effect on endosomal acidification (Figure 6D). With these experiments, we could show that rNleC, to some extent, is indeed degraded by lysosomes. However, cleavage of p65 after incubation with stimulated whole cells (Figure 2D) and separation of rNleC from lysosomes after 24 h indicate that at least some rNleC molecules can escape from the endo-lysosomal compartment.

### rNleC escapes from endosomes

**Figure 7:**
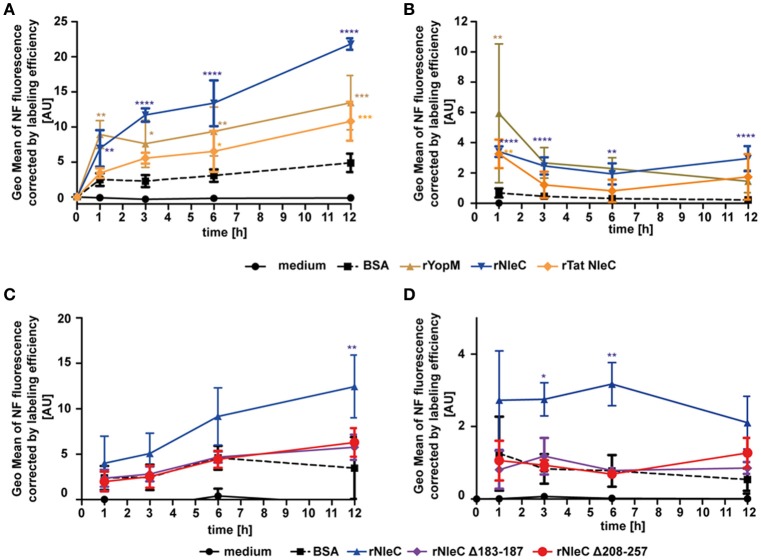
**rNleC escapes the endosomes and reaches the cytosol**. **(A,C)** 25 μg/ml NF-labeled proteins were incubated for the indicated times at 37°C, washed and quenched using acidic buffer. Fluorescence intensities of acid-quenched and trypsinized cells were measured using flow-cytometry. Depicted is the GEO Mean of NF-fluorescence corrected by labeling efficiencies and are the mean ± SD of three independent experiments is given and the levels of significance (two-way ANOVA, followed by Bonferroni's multiple comparison test) compared to BSA are indicated (see below). **(B,D)** Pulse-chase experiments: 50 μg/ml NF-labeled proteins were incubated with pre-chilled cells for 30 min at 4°C, followed by incubation at 37°C for 1 h. Media was exchanged and cells were incubated for the indicated time at 37°C, washed and quenched using acidic buffer. Fluorescence intensities of trypsinized cells were measured using flow-cytometry. Depicted is the GEO Mean of NF-fluorescence corrected by labeling efficiencies. Data are presented after subtraction of background fluorescence, correction by labeling efficiencies and are the mean ± SD of three independent experiments. The levels of significance (two-way ANOVA, followed by Bonferroni's multiple comparison test) compared to BSA are indicated. ^*^p ≤ 0.05, ^**^p ≤ 0.01, ^***^p ≤ 0.001, and ^****^p ≤ 0.0001.

To elucidate whether rNleC can escape from the endosomal-lysosomal pathway to reach its target in the cytosol as it had been shown for rYopM (Rüter et al., 2010), experiments with naphthofluorescein (NF)-labeled proteins were conducted. NF is a pH-sensitive dye, which emits fluorescence signals in neutral environments, whereas its fluorescence is quenched in acidic environments. Thus, NF-labeling of proteins can be used to measure only those proteins that have reached the neutral environment of the cytosol or the nucleus, whereas the fluorescence signal is quenched in an acidic environment as for example in late endosomes or lysosomes (Qian et al., 2015). To verify whether this fluorescent dye was a suitable marker for delivery of proteins into the cytosol, we incubated cells with NF-labeled BSA, which is endocytosed but subsequently degraded in lysosomes (Humphries et al., 2012). We could only measure background fluorescence when cells were treated with NF-BSA, whereas we could observe a significant increase in fluorescence after artificially disrupting endosomes with chloroquine (Figure S9). Thus, NF-BSA is a suitable control for a protein that is endocytosed but is not delivered into the cytosol. To follow uptake into the neutral environment (e.g., the cytosol) over time, NF-labeled proteins were added to cells for 1, 3, 6, and 12 h and uptake was measured by monitoring increasing fluorescence using flow cytometry (Figure 7A). During 12 h of incubation fluorescence of NF-BSA increased only very little in comparison with the NF-labeled rYopM, rNleC, and rTat NleC proteins. The strongest uptake could be observed for rNleC alone. This is in line with previous results, where also a reduced uptake for rTat NleC compared to rNleC was found (Figure 3A). In this experimental set-up, NF-labeled proteins were incubated with the cells during the complete incubation time, which means that proteins could also be continuously introduced into the cells. Since early endosomes are only slightly acidic and thus NF-labeled proteins residing in early endosomes might also emit fluorescence, we performed pulse-chase experiments to account for a putative continuous uptake of proteins. In this way, we were able to exclude a contribution of fluorescence signals derived from proteins residing in early endosomes. In the pulse-chase assays we confirmed for all time points higher fluorescence values for the NF-labeled rNleC, rYopM, and rTat NleC proteins compared to NF-labeled BSA. This indicated that rNleC, rYopM, and rTat NleC were able to escape from endosomes (Figure 7B). After 1 h, a reduction in fluorescence signals was observed which might be either due to degradation of proteins or might be caused by the maturation of early and non-acidified endosomes to late and acidified endosomes. Yet, the fluorescence signals remained at the same level between 3 h and 12 h suggesting that these proteins, once reaching the neutral environment, were stable within the cell and were not further degraded. To clarify the intracellular behavior of the deletion mutants, we performed the NF-uptake assay (Figure 7C) and the pulse-chase experiment (Figure 7D) with NF-labeled NleC deletion mutants and observed similar fluorescence intensities as for NF-labeled BSA, indicating that these proteins were apparently unable to be delivered into the cytosol.

### pH-induced conformational changes of rNleC precede endosomal-lysosomal escape

**Figure 8:**
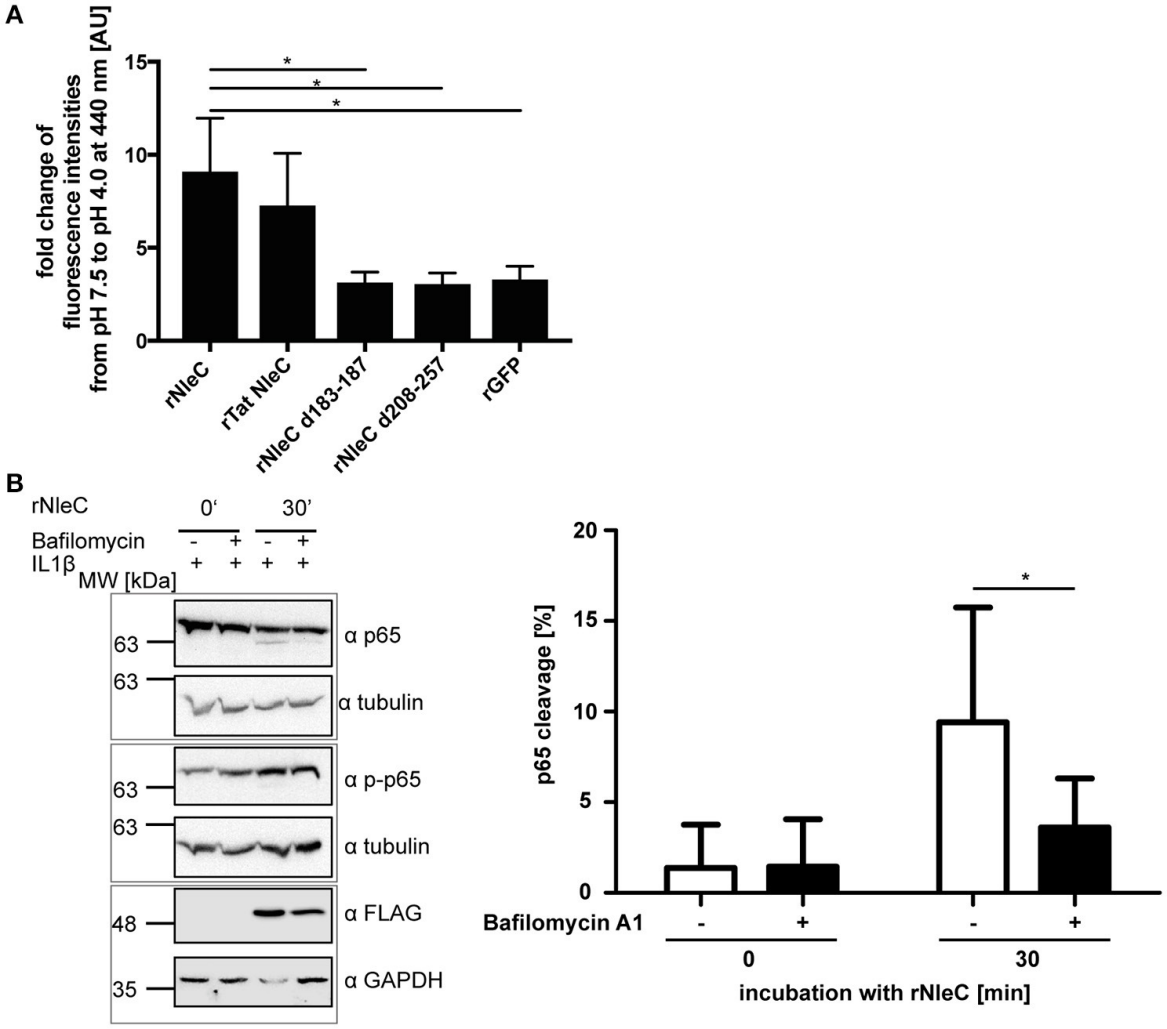
**rNleC escapes endosomes upon structural rearrangements at low pH. (A)** Fold change in TNS fluorescence of rNleC, rNleC variants, and rGFP between pH 7.5 and pH 4.0. 1.5 μM recombinant protein was incubated with 150 μM TNS for 15 min at RT. Data are presented after subtraction of background fluorescence, followed by calculation of the fold change in TNS fluorescence at 440 nm and are the mean ± SD of three independent experiments. The statistical significance (one-way ANOVA, followed by Bonferroni's multiple comparison test) compared to rNleC is indicated (see below). **(B)** HeLa cells were pre-incubated for 2 h with 10 nM Bafilomycin A1 followed by incubation with 25 μg/ml rNleC for 30 min at 37°C. Cells were stimulated with 10 ng/ml IL1β for 20 min at 37°C. Cells were lysed in RIPA buffer for 30 min at 4°C. Cleavage of p65 was analyzed by Western blotting using α-p65 antibodies. Tubulin was used as loading control. gray boxes indicate bands from the same gel. The bar graph shows the densitometric evaluation of immunoblots of at least three independent experiments (mean ± SD) with level of significance (two-way ANOVA, followed by Bonferroni's multiple comparison test) indicated. ^*^p ≤ 0.05.

NleC is structurally and functionally homologous to the A subunit of the zinc-metalloprotease A-B toxin AIP56 of (*Phdp*). AIP56 is the major virulence factor for colonization and dissemination in sea bass, the natural host for *Phdp*. Just like NleC, AIP56 also harbors a HEXXH motif, which is essential for cleaving its target, NF-κB p65. However, in contrast to NleC, AIP56 does not down-regulate pro-inflammatory cytokines, such as TNFα, IL1β, or IL8, but leads to apoptosis (Do Vale et al., 2005; Silva et al., 2013; Pereira et al., 2014). Besides the sequence homology of NleC and the A subunit of AIP56 including the catalytic center and their identical cellular target protein (Figure S10A), NleC and the A subunit of AIP56 also share a remarkable similarity in their predicted 3D-structure (Figure S10B). AIP56 induces its endosomal escape due to structural changes upon acidification within the endosomes (Pereira et al., 2014). Since NleC and the A subunit of AIP56 are also quite similar regarding the distribution of polar and hydrophobic amino acids (Figure S10C), we asked whether alterations in pH could lead to conformational changes in rNleC, which would promote its escape from acidified endosomes/lysosomes. As an indicator for conformational changes induced by acidic pH we used the hydrophobic molecular probe 6,P-toluidinyl-naphtalene-2-sulphonate (TNS) (Golczak et al., 2001; Albani, 2009) and monitored its alterations in fluorescence between pH 7.4 and pH 4.0. TNS is a molecular probe that is quenched in hydrophilic environments, whereas it emits fluorescence in hydrophobic environments. Hence, conformational changes resulting in increasingly exposed hydrophobic surfaces would increase binding of TNS and also fluorescence intensities. For easier comparison, alterations in TNS fluorescence have been depicted as fold changes at 440 nm (Figure 8A and Figure S11). Interestingly, rNleC as well as rTat NleC showed remarkably high fold changes in TNS fluorescence, whereas the rNleC deletion constructs exhibited fold changes in TNS fluorescence comparable to rGFP. This suggests that acidification of lysosomes induces substantial alterations in the conformation of rNleC and thus might be instrumental for rNleC to escape from endosomes, which is reminiscent of AIP56. Furthermore, these results might explain why deletions in NleC result in an abrogated escape from endosomes compared to wt rNleC. To assess whether acidification is the underlying mechanism facilitating endosomal escape of rNleC, we blocked endosome acidification with Bafilomycin A1 in HeLa cells for 2 h. Next, the cells were incubated with rNleC for 30 min and subsequently stimulated with IL1β to activate NF-κB signaling. As readout, the cleavage of p65 was determined by Western blotting (Figure 8B). Under these conditions the cleavage of p65 was reduced by 45%, which suggests that the acidification of endosomes is an important step for rNleC to reach its intracellular target p65.

### Evolutionary link between the T3SS effector protein NleC and bacterial toxins

**Figure 9:**
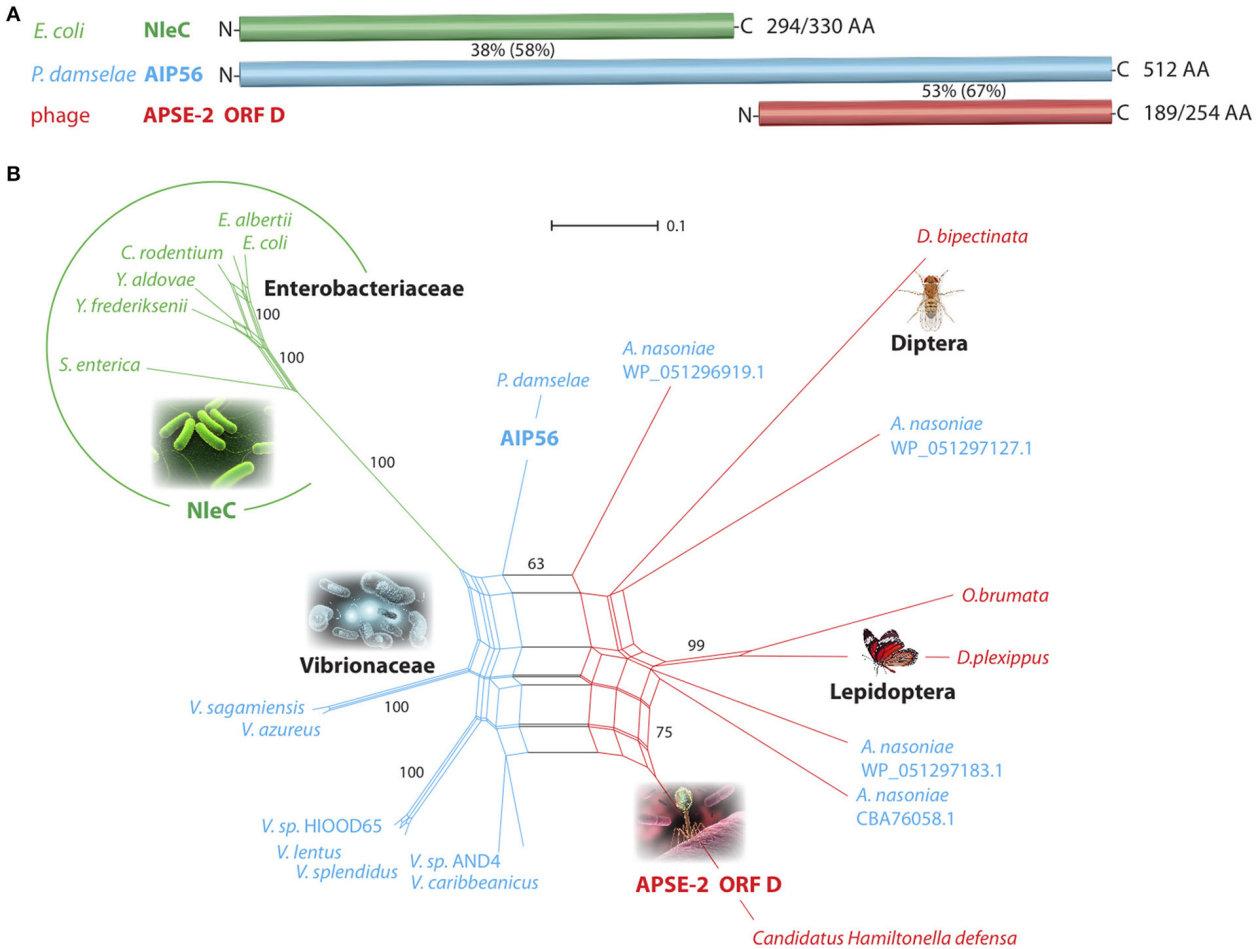
**Affiliations of NleC/AIP56/APSE-2 ORF D-like amino acid sequences**. **(A)** Schematic representation of the toxin-associated peptide regions from *E. coli* (NleC), *P. damselae* (AIP56), and the bacteriophage APSE-2 (ORF D). Percentiles represent the amino acid identity (in parentheses similarity) between corresponding sequence regions. The numbers of aligned/total amino acids are placed to the right of the bars. N- and C-termini are indicated. **(B)** Network phylogenetic analysis. The NleC-encoding *Enterobacteriaceae* are circled (green). The blue parts of the net represent AIP56-coding *Vibrionaceae*, and the red parts *Enterobacteriaceae* and *Eukaryonta* sequences that show similarity to the APSE-2 OFR D bacteriophage amino acid sequence. Based on this similarity, *A. nasoniae* was placed in the red part of the net; however, it contains an AIP56-like sequence (shown in blue letters). The reproducibility of selected areas of the net investigated by a bootstrap test (10,000 replications) show NleC-encoding representatives 100% separated from other sequences. The scale bar represents substitutions/site. Genera abbreviations for *Enterobacteriaceae*: *Escherichia, Yersinia, Salmonella, Arsenophonus*; *Vibrionaceae*: *Vibrio, Photobacterium*; *Eukaryonta: Drosophila, Operophtera, Danaus*.

The uptake of rNleC into endosomes and the subsequent escape from endosomes into the cytosol displays features known from short-trip A-B toxins. Because NleC and the A subunit of the short-trip A-B toxin AIP56 of *Photobacterium damselae* subsp. *Piscicida* (*Phdp*) show a high sequence similarity, we investigated their evolutionary relationship in more detail. A blastp search indicated that the *E. coli* NleC metalloprotease exhibits 38% identity (58% similarity) to the 294-amino acid N-terminal part of the plasmid-encoded apoptosis gene product, AIP56 of *Phdp* (Figure 9A and Data Sheet 1). This strongly suggests a potential relationship of these two bacterial virulence factors that both target, albeit by different mechanisms, the NF-κB signaling system. The 189 amino acid C-terminal region of AIP56 has 53% identity (67% similarity) to the γ-proteobacterium *Candidatus Hamiltonella defensa* (*Entero-bacteriaceae*) genome-integrated prophage product APSE-2 ORF D (*A. pisum* secondary endosymbiont; CP001277.1; position 1520562–1521326; Figure 9A). The annotated hypothetical protein D is 100% identical to the corresponding region in the isolated bacteriophage genome product APSE-2 (NC_011551.1; position 7577–8341; *A. pisum* secondary endosymbiont phage; Moran et al., 2005). A possible association of the N-terminal domain of AIP56 to NleC and the C-terminal domain to a hypothetical protein of the bacteriophage APSE-2 had been proposed previously by Silva et al. (2013).

In an expanded blastp screening of the NCBI non-redundant protein sequences using NleC, AIP56, and APSE-2 ORF D AA query sequences, we retrieved significant hits for diverse *Enterobacteriaceae* and *Vibrionaceae*. Remarkably, along with the bacterial and phage hits, some *Eukaryonta* were also represented with annotated hypothetical proteins similar to the C-terminal AIP56/APSE ORF D query sequences, with the highest similarity to the sequences of APSE-2 ORF D (46–60%). To visualize the relationships of these hits, we derived an evolutionary network (Figure 9B) that shows a clear, significantly separated cluster of the NleC-coding *Enterobacteriaceae* (green; bootstrap value 100%). AIP56-like sequences are dispersed over many *Vibrionaceae* including diverse species from the genus *Vibrio* as well as from *P. damselae* and the *Enterobacteriaceae Arsenophonus nasoniae* (blue species names in Figure 9B). Eukaryotic representatives as well as the bacteriophage APSE-2 sequence and *Arsenophonus* sequences are connected in the red section of the net.

## Discussion

**Figure 10:**
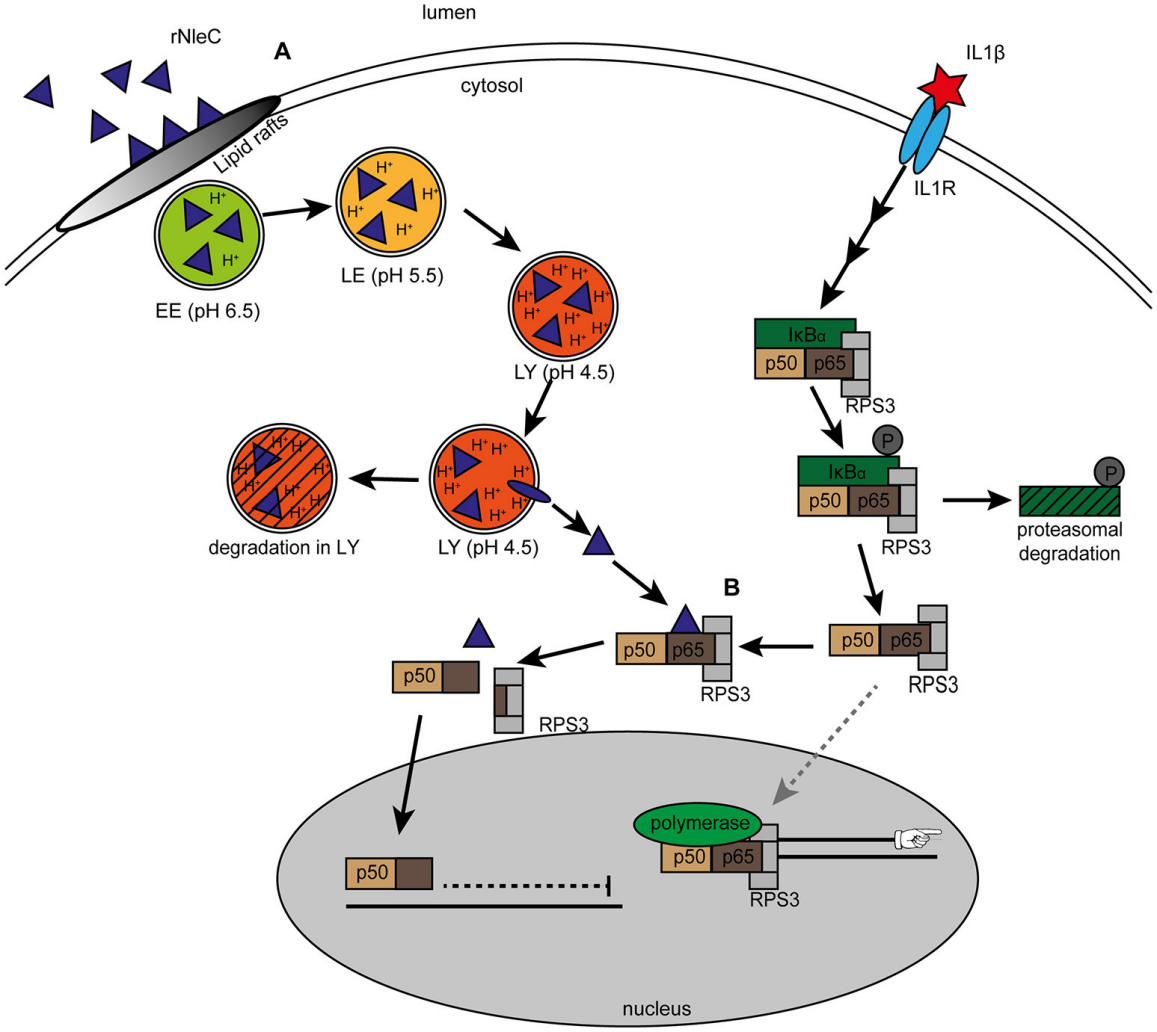
**Uptake and intracellular trafficking of rNleC. (A)** rNleC enters eukaryotic cells via lipid rafts and follows the endosomal-lysosomal pathway. Upon acidification of endosomes, rNleC performs a change in its three-dimensional structure and escapes from endosomal compartments into the cytosol. (EE: early endosomes, LE: late endosomes, LY: lysosomes). Protein that does not escape from endosomes is degraded. **(B)** Cytosolic rNleC only cleaves NF-κB p65 when the NF-κB signaling pathway is activated (for example by IL1β). Cleavage of p65 results in a C-terminal p65 fragment bound to p50, which translocates into the nucleus and an N-terminal fragment bound to RPS3, which remains in the cytosol. Without the specifier RPS3, RPS3-depent NF-κB genes such as the pro-inflammatory cytokines IL1β, IL8, or TNFα are not transcribed and innate immune responses are dampened.

In this study, we characterized the EPEC effector NleC as an autonomously cell-entering effector protein and demonstrated that (rNleC) exhibits structural and functional similarities to the A subunit of the short-trip exotoxin AIP56 derived from (*Phdp*), a major fish pathogen. rNleC enters eukaryotic cells independent of a T3SS or any other bacterial factor following the endocytic pathway. Upon acidification of endosomes, rNleC undergoes changes in its conformation, allowing for a subsequent endosomal escape (Figure 10A). Afterwards rNleC reaches and cleaves p65 of the NF-κB pathway–interestingly only in cells where the NF-κB pathway had been previously activated by IL1β (Figure 10B). Although autonomously taken-up by host cells, rNleC does not fulfill important criteria for a cell-penetrating effector (CPE).

T3SS-injected NleC has been reported to cleave p65 of NF-κB at the N-terminus (Mühlen et al., 2011; Giogha et al., 2015) and processing has been described to occur in the Rel-domain between Cys38 and Glu39 (Giogha et al., 2015). Furthermore, it has been proposed that the cleaved 38 aa p65 peptide binds to RPS3 and subsequently hinders NF-κB and RPS3 interactions thereby reducing the binding of NF-κB to RPS3-dependent κB-binding sites. This abrogates transcription of pro-inflammatory cytokines (Wier et al., 2012; Hodgson et al., 2015).

As our initial *in silico*-analysis predicted two potential PTDs (CPPs) in the sequence of NleC (Figure 1) we expressed NleC to characterize the cell-penetrating and functional abilities of isolated (rNleC). We found the isolated recombinant rNleC to be functional as p65 of NF-κB was cleaved in cell lysates (Figure 2A). However, given that the cleavage could not be detected in intact cells, we hypothesized that during infection and T3SS-dependent delivery of effector proteins the NF-κB pathway would usually be activated which might not be the case with isolated and purified rNleC. Hence, we incubated target cells for activation with IL1β after addition of rNleC and analyzed p65 by Western blotting. As depicted in Figure 2D in intact HeLa cells with an activated NF-κB pathway we could clearly demonstrate processing of p65. The requirement for an activated NF-κB pathway was further confirmed by using the inhibitor BAY 11-7085 which abrogated the effect of IL1β (Figure 2E). NleC-dependent cleavage of p65 only from an activated NF-κB signaling cascade leads to more specificity. During EPEC infection, the apparent prerequisite for an activated NF-κB pathway restricts cleavage of p65 to those cells or even to molecules that are taking part in the immune response. This circumvents side effects of random p65 cleavage that would impact proliferation, differentiation or apoptosis, which might negatively influence the prolongation of infection.

These findings confirmed not only the functionality of rNleC in processing p65 in activated target cells but, furthermore, also demonstrated that rNleC reached its target protein in the cytosol. This is reminiscent of other cell-penetrating effector proteins (CPE) previously characterized by our group, such as rYopM or rSspH1 (Rüter et al., 2010; Lubos et al., 2014), which autonomously enter target cells via CPP-mediated endocytic uptake followed by endosomal escape (Scharnert et al., 2013).

Next, we addressed possible uptake mechanisms for rNleC and, to confirm the functionality of the two predicted PTDs, we analyzed cellular uptake of several NleC variants (Figure 3) and of the predicted PTD sequences alone or in combination with GFP (Figure 4) by FACS analysis and fluorescence microcopy (Figure 3C). We clearly demonstrated cellular entry of rNleC and of rTat NleC (Figures 3A,C). Although it appeared that the presence of these predicted PTDs was indeed necessary for full uptake efficiency as shown for the variant rNleC Δ208–257 lacking both PTDs (Figure 3D), the predicted PTDs themselves were unable to enter cells, and, furthermore, failed in cargo transport (Figure 4). CPPs are defined as short peptide sequences able to translocate cargo across eukaryotic membranes (El-Andaloussi et al., 2007; Choi et al., 2012; Bechara and Sagan, 2013), a property, which we could exclude for the predicted PTDs in NleC (Figure 4). Therefore, rNleC does not qualify as a cell-penetrating effector or CPE, such as rYopM (Rüter et al., 2010) or rSspH1 (Lubos et al., 2014). Interestingly, the rNleC variant displaying the Tat CPP at its N-terminus (Figure 1B) entered cells to a significantly lower degree than the authentic rNleC (Figure 3A). Moreover, also uptake of a deletion variant of rNleC lacking only five amino acids of the HEXXH Zn-binding motif (NleC Δ183–187) was reduced substantially (Figure 3D). All investigated proteins were not cytotoxic according to the LDH cytotoxicity assay (Figure S3).

Next, we investigated whether endocytosed rNleC, such as rYopM or rSspH1 would follow the endocytic transport pathway. Therefore, we investigated the effects of several inhibitors on cell entry of rNleC (Figure 5A). Only methyl-β-cyclodextrin (MβCD), which affects pathways involving lipid rafts, showed some effect. Co-localization with early endosomes (Rab5), late endosomes (Rab7), and early lysosomes (CD63) was demonstrated by fluorescence microscopy (Figure 5C). However, we could not detect any co-localization of rNleC with markers indicative of retrograde trafficking, such as Rab6, COP1, and GM130 (Figure S6B) (Nakamura et al., 1995; Grigoriev et al., 2007; Beck et al., 2009) and, therefore, excluded this pathway as a possible mechanism for cytosolic entry of rNleC. Retrograde trafficking from endosomes to the ER is an important mechanism of long-trip A-B toxins, such as Shiga toxin (Sandvig and van Deurs, 1996) or cholera toxin (Wernick et al., 2010) to escape from endosomes and finally reach their intracellular cytosolic targets (Sandvig and van Deurs, 2005).

Since we visualized co-localization of rNleC with early lysosomes (CD63), we investigated the functionality of lysosomes by using Lysotracker red DND99, a pH-dependent dye targeting lysosomes (Figure 6B), since acidification is necessary for lysosomal function. Here, we monitored co-localization of rNleC with acidified lysosomes between 4 and 16 h of incubation. Co-localization increased over time in parallel to the increase of intracellular localization of rNleC. Interestingly, co-localization of rNleC with Lysotracker red DND99 decreased between 16 and 24 h and, in parallel, the previously particulate fluorescence of rNleC became more diffuse indicating that rNleC was separated from lysosomes between 16 and 24 h putatively indicating a translocation into the cytosol (Figures 6B,C).

To further confirm that rNleC escapes from endosomes we monitored the fluorescence of naphthofluorescein (NF)-labeled rNleC including the rTat NleC variant compared to rYopM. The NF fluorophore is quenched at low pH, such as in acidified endosomes, and only emits fluorescence at neutral pH as in the cytosol. We compared the cytosolic delivery of rNleC and rTat NleC to that of the CPE rYopM which has been described to escape from endosomes after being endocytosed (Scharnert et al., 2013). In these experiments BSA served as a negative control, because following endocytosis, it is degraded in lysosomes and never enters the cytosol (Humphries et al., 2012). The delivery of rNleC into the cytosol was even higher compared to the established cell-penetrating effector rYopM, indicating an endosomal escape (Figure 7). Furthermore, the fluorescent signal remained stable for up to 12 h in a pulse-chase assay indicating substantial stability of the rNleC protein once it reached the cytosol.

Endosomal escape is an established concept, which has been amply described for many bacterial A-B toxins (Sandvig and van Deurs, 2005; Williams and Tsai, 2016) and also for the recently identified CPE YopM (Scharnert et al., 2013). In general, two different endosomal escape mechanisms have been observed for A-B toxins. Long-trip A-B_(5)_-toxins, such as Shiga toxin, pertussis toxin or cholera toxin, are routed through the trans-Golgi network and are released from the endoplasmic reticulum into the cytosol (Sandvig and van Deurs, 1996; Johannes et al., 1997; Majoul et al., 1998; Dixit et al., 2008; Capitani and Sallese, 2009; Sandvig et al., 2010; Wernick et al., 2010). Short-trip toxins, such as clostridial toxins, anthrax or diphtheria toxin translocate already across endosomal membranes (Barth et al., 2000; Puhar et al., 2004; Liu et al., 2014). Here, conformational changes upon acidification trigger endosomal escape.

NleC has striking similarities to the A subunit of AIP56, an A-B toxin from *Phdp* (Figure S10). Unlike NleC, AIP56 is not translocated into the cytosol via a T3SS during infection, but is taken up by clathrin-mediated endocytosis (Pereira et al., 2014). As common for most A-B toxins, the catalytic effect and the uptake-inducing signal is located in distinct domains of the protein, in which the N-terminus (A subunit) has the catalytic function and the C-terminus (B subunit) is responsible for receptor-mediated endocytosis (Pereira et al., 2014). Nevertheless, the A subunit of heat-labile enterotoxin was shown to have cell-penetrating functions as well (Liu et al., 2016). Once endocytosed, AIP56 co-localizes with early endosomes. The acidification of the endosomes leads to a conformational shift in AIP56, which in turn induces its endosomal escape (Pereira et al., 2014). Hence, AIP56 qualifies as a short-trip A-B toxin like diphtheria toxin (Papini et al., 1993). Similar to AIP56, rNleC also follows the endosomal-lysosomal route after entering the cell and escapes from endosomes following acidification. Interestingly, we showed that both NleC deletion variants exhibit a reduced capability to perform conformational changes at low pH (Figure 8A).

Hence, we hypothesized that acidification and the ensuing structural changes in rNleC trigger the endosomal escape mechanism of rNleC. Consequently, inhibition of endosome acidification with Bafilomycin A1 would also abolish rNleC-dependent p65 cleavage (Figure 8B). This indicates that rNleC like AIP56 requires conformational changes induced by low pH in acidified endosomes-lysosomes to escape from these compartments and to reach the cytosol.

Most virulence factors of pathogenic bacteria that target the NF-κB signaling cascade are translocated into the host via T3SS. Although at present it is not known whether *Phdp* might express a T3SS, the cytosolic delivery of the Zn-metalloprotease AIP56 has been described to follow the endocytic uptake route of A-B toxins. It has been suggested that the fusion of the A and B subunits occurred following horizontal gene transfer, since the B subunit is reminiscent of a protein of unknown function from *Acrythosiphon pisum* bacteriophage APSE-2 (Degnan et al., 2009).

Investigating possible evolutionary linkages of NleC and AIP56 we found that its amino acid sequences merge into one *Enterobacteriaceae* cluster significantly separated from all other investigated species (100% bootstrap support; Figure 9B). The amino acid sequence identities in this cluster range from 70 to 94% (similarity 80–96%). The high affinities of NleC-related sequences clearly show their common origin inside *Enterobacteriaceae*. However, the significantly closer relationship of NleC amino acid sequences of *E. coli* to *Y. aldovae* and *Y. frederiksenii* compared to *S. enterica* as well as missing NleC genes in other related species or strains let us assume that, besides vertical transmission, lateral gene transfer is involved in the shape of the presented network and the evolution of the suppression of inflammatory host responses.

The similarity of NleC and the N-terminus of AIP56 and the location of NleCs inside a heterogenous *Vibrionaceae* cluster suggest an evolutionary origin of NleC from an AIP56-like source. AIP56-like amino acid sequences are distributed in two genera of the *Vibrionales*, Vibrio and Photobacterium, and in the *Enterobacteriaceae Arsenophonus*, another heritable bacterial endosymbiont of a broad range of insects, such as *Hemiptera, Hymenoptera*, and *Diptera*.

Interestingly, genome sequencing of the *H. defensa* host and associated APSE-2 bacteriophage revealed an identical 254 amino acid-long toxin-like sequence (ORF D) encoded by both genomes, and 189 of their amino acids show strong similarity (67%) to the C-terminus of AIP56. This similarity makes it conceivable that AIP56s have evolved after a phage infection and chromosomal bacterial integration, similar to that shown for *H. defensa* (above), in diverse *Vibrionaceae*. The NleC system may have been a next step in evolving complex toxin injection systems. An APSE protein D-like-encoding hypothetical gene was also detected in the genomes of the butterfly *Danaus plexippus*, the moth *Operophtera brumata*, and diptera *Drosophila bipectinata*, inherited via a thus far unknown vector and with unknown function. Because the sequences are most interconnected to *A. nasonia* sequences (Figure 9B) we suggest a transfer via an *Arsenophonus*-APSE like system.

Based on these arguments we propose the following evolutionary scenario. A bacteriophage, such as APSE-2, containing a protein ORF D-like toxin sequence in its arsenal of gene products, which is actually intended as a *guest-present* to its bacterial host, infects for example *H. defensa*. However, the bacteria themselves cannot exploit the toxin directly, but after chromosomal integration of the phage-born toxin gene, as just shown here for *H. defense*, intensive evolving/recombination/lateral transfer possibly converted it into an AIP56-like toxin (e.g., as present in *P. damselae*). Perhaps also derived from the same toxin gene conversion into a precursor of the protease NleC in coevolution with the complex T3SS injection machinery occurred. The latter system might have even evolved in the potential common ancestor of the NleC-presenting *Enterobacteriaceae*. The toxin originally transferred to the eukaryotic host might have (1) protected the host, for example, from a parasitic attack of wasps, as realized in aphids via *H. defense*, and/or (2) paralyzed the host's inflammatory response after bacterial infection. Naturally, this scenario presents just one conceivable evolutionary pathway that requires further intensive investigations to find missing vectors and evolutionary intermediates.

Our finding that rNleC can overcome eukaryotic membranes autonomously and independent of the T3SS in combination with the remarkable sequence homology to the A subunit of AIP56 suggests that NleC and AIP56 evolved from a common ancestral virulence protein. These interesting results are also relevant for other effector proteins being able to translocate autonomously across eukaryotic membranes, such as YopM from *Yersinia enterocolitica*, SspH1 from *Salmonella typhimurium* and TcpC from uropathogenic *E. coli* (UPEC) (Rüter et al., 2010; Yadav et al., 2010; Snyder et al., 2013; Lubos et al., 2014). The relevance of this property during bacterial infections has not been determined and is under investigation. Potentially, these virulence factors evolved from single acting proteins to bundled effectors under the control of the T3SS machinery during evolution. The translocation of proteins by T3SS is considered to be very efficient since this has several advantages over the release of toxins into the environment. As the T3SS injects proteins directly and without intermediate steps into the cytosol, they are not endangered by dilution or degradation during an intermediate extracellular state. In addition, they are also not prone to be degraded by lysosomes when taken up via the endocytic pathway or to be excreted by recycling endosomes (Pereira et al., 2014). Furthermore, injection by the T3SS protects against neutralizing antibodies, which would strongly dampen bacterial virulence and thus bacterial replication. Taken together, translocating effectors with a T3SS instead of environmental release followed by endocytic uptake appears to be a more economic and largely optimized virulence strategy. It can thus be argued that the self-delivering abilities of (some) effector proteins do not play a major role in infections by today's bacteria, but are an evolutionary artifact from times before T3SS had evolved. However, T3SS injection requires cellular attachment of the pathogen and thus does not directly affect nearby host cells. Although the translocation of T3SS effectors into the cytosol of eukaryotic cells is described as tightly regulated and absolutely dependent on cell-cell contact (Lee and Galán, 2004), T3SS secretion of effectors into the culture medium can also occur. Induced by environmental factors, such as temperature, calcium and sodium chloride concentrations, effectors might also be secreted via the flagellum of bacteria (Young and Young, 2002; Lee and Galán, 2004). Examples for additional flagellar secretion include *Salmonella typhimurium* effectors SopE and SptP (Lee and Galán, 2004). The extra-T3SS secretion—affecting also bystander cells of the host—might be a mechanism to down-regulate immune responses in parallel or even before bacteria are able to attach and translocate the effectors via the T3SS. Since the SPI2-T3SS of *Salmonella typhimurium* and EPEC T3SS as well as some effectors are closely related (Hensel et al., 1998), a flagellar export of effectors of EPEC does not seem unlikely.

In this study, we have identified and characterized the type 3 secretion system-dependent effector protein NleC, which—as a recombinant expressed, isolated and purified protein—can overcome cellular membranes independently of a T3SS or other bacterial factors in a manner resembling short-trip A-B toxins. The protein is endocytosed and able to escape into the cytosol upon acidification of endosomes. Cytosolic rNleC cleaves its target NF-κB p65 requiring prior activation of the NF-κB signaling cascade, thereby restricting its activity specifically to stimulated cells. Since other studies revealed the function of NleC either in the context of bacterial infections, where the NF-κB pathways is naturally stimulated (Baruch et al., 2011), or as ectopically expressed proteins, where the amount of rNleC produced exceeds that in natural translocation (Mühlen et al., 2011), the preference for p65 upon pathway stimulation was not detected previously. It has also been discussed earlier that the resulting N-terminal 38 amino acid segment of the p65 cleavage binds to the NF-κB specifier RPS3 (Hodgson et al., 2015), targeting the latter to NF-κB/RPS3-dependent transcription of cytokine genes (Wier et al., 2012). Interference with p65-RSP3 interactions ultimately leads to a reduced transcription of pro-inflammatory cytokines. This might also explain, why NleC-dependent p65 cleavage is specific for the reduction of pro-inflammatory cytokines and does not affect differentiation and proliferation, which are also pathways regulated by NF-κB. These properties might identify rNleC as an interesting candidate for future applications, e.g., as a potential therapeutic against immune disorders, as it shuts down pro-inflammatory reactions in a non-stoichiometric manner.

## Author contributions

Conceived and designed the experiments: AS, SN, CR, and MAS. Performed the experiments: AS, BK, LL, MF, and SN. Analyzed the data: AS, SN, JS, CR, and MAS. Wrote the paper: AS, CR, JS, and MAS.

## Funding

This project was supported by the Deutsche Forschungsgemeinschaft [SFB1009 TP B03; DFG RU 1884/2-1; Graduiertenkolleg GRK1409, and the Cells-in-Motion Cluster of Excellence (EXC 1003–CiM)]. The funders had no role in study design, data collection and analysis, decision to publish, or preparation of the manuscript.

### Conflict of interest statement

The authors declare that the research was conducted in the absence of any commercial or financial relationships that could be construed as a potential conflict of interest.

## Supplementary material

Figure S1 **(A)** Arginines are enriched in predicted PTDs. The number of arginine residues was counted. Arginine residues per sequence in % (left column) and arginine residues per sequence length in % (right column) were calculated. Arginines are enriched in predicted PTDs. **(B)** Catalytic activity of rNleCH187Y is not abrogated. HeLa cells or HeLa cell lysates were incubated with 125 μg recombinant proteins (in whole cells this represents 25 μg/ml). Samples were subjected to Western blotting and p65 cleavage was detected using α-p65 antibodies.

Figure S2 **IL1β does not permeabilize cells**. HeLa cells were incubated with 50 μg/ml rNleC for 30 min together with 1 μg/ml PI. Cells were stimulated with10 ng/ml IL1β (black bars) for 3.5 h or not (white bars), trypsinized and PI uptake was measured using flow cytometry. The bar graph shows the results of three independent experiments (mean ± SD).

Figure S3 **Recombinant proteins are not cytotoxic**. The percentage of released LDH was measured to exclude a cytotoxic effect of rNleC or of the rNleC variants on HeLa cells. Experiments are presented as the mean ± SD of three individual experiments.

Figure S4 **IL1β does not influence the uptake of rNleC or rTat NleC**. HeLa cells were incubated with 50 μg/ml protein for 30 min. Cells were stimulated with 10 ng/ml IL1β (black bars) for 3.5 h or not (white bars) and trypsinized. Trypsinized cells were quenched with Trypan Blue (final concentration 0.2%) and fluorescence was subsequently measured by flow cytometry. The bar graph shows the results of three independent experiments (mean ± SD).

Figure S5 **PTD1 and PTD2 are not CPPs**. Fluorescence microscopy of HeLa cells incubated with 576 nM FITC-labeled PTD1, PTD2, Tat and NPP (green) for the indicated times. PFA-fixed cells were stained with Phalloidin TRITC (red) and Draq5 (blue). Fluorescence images were generated using a Zeiss LSM 510 microscope. Z-stacks of single cells were taken.

Figure S6 **(A)** Endocytic inhibitors do not inhibit catalytic activity of rNleC. HeLa cell lysates were incubated with the indicated endocytosis inhibitors (Dynasore 30 mM, Cytochalasin D 200 μM, Amiloride 19 mM, Filipin 3.8 mM, Nocodazole 16.5 mM and MβCD 50 mM) together with rNleC (50 μg/ml) for 30 min at 4°C. Cleavage of p65 was analyzed by Western blotting using α-p65 antibodies. FLAG-tagged rNleC, was detected with an α-FLAG antibody. Tubulin and GAPDH were used as loading controls. gray boxes indicate bands obtained with the same gels. **(B)** rNleC does not co-localize with markers of retrograde trafficking. HeLa cells were pre-incubated with FITC-labeled rNleC for the indicated times. Cells were fixed, quenched, permeabilized and immunostained with α COP1, α Rab6 or α GM130 antibodies and were visualized with a Cy3-labeled secondary antibody (red). Nuclei were stained with DAPI (blue). Fluorescence images were generated using a Zeiss LSM800 microscope. The scale bar represents 10 μm.

Figure S7 **PI uptake of endocytosis inhibitor treated cells**. Adherent HeLa cells were pre-incubated with the indicated endocytosis inhibitors for 1 h and treated with FITC-labeled rNleC (50 μg/ml) for 4 h at 37°C. PI fluorescence was subsequently measured by flow cytometry. The bar graph shows the results of at least three independent experiments (mean ± SD).

Figure S8 **Bafilomycin A1 blocks acidification of lysosomes**. HeLa cells were incubated with 10 nM Bafilomycin A1 for the indicated times. 3 h prior to the end of the experiment 400 nM Lysotracker DND99 was added to the supernatant. Cells were trypsinized and the amount of acidified endosomes was measured using flow cytometry. The data are presented after subtraction of background fluorescence and are the mean ± SD of three independent experiments. ^***^*p* ≤ 0.001.

Figure S9 **Chloroquine releases endocytosed BSA into the cytosol**. HeLa cells were incubated with 0, 150 or 300 μM Chloroquine together with 25 μg/ml BSA for 3 h. Cells were washed and quenched with 0.2 M glycine abrogate signals from the outside of the cell. Geo mean of FL3 fluorescence corrected by labeling efficiency is depicted. Bar graph represents the mean of at least three independent experiments (mean ± SD) with the level of significance indicated (one-way ANOVA, followed by Bonferroni multiple comparison test). ^**^*p* ≤ 0.01.

Figure S10 **Sequential and structural homologies of NleC and AIP56. (A)** Sequence alignment of the A subunit of AIP56 and NleC. **(B)** Overlay of structure predictions for NleC (blue) and the A subunit of AIP56 (yellow) with Swiss-PdbViewer 4.1.0. The catalytic domain (HEXXH) motif is marked in orange (NleC) or green (AIP56). **(C)** Hydrophobic amino acids in the sequence of NleC (blue) and the A subunit of AIP56 (yellow) are marked in green using Swiss-PdbViewer 4.1.0.

Figure S11 **Conformational changes at low pH**. Complete spectra of TNS fluorescence of rNleC, rNleC variants and rGFP at pH 7.5, 7.0, 6.5, 6.0, 5.5, 5.0, 4.5, and 4.0. Data are presented after subtraction of background fluorescence and are the mean ± SD of three independent experiments.

Data Sheet 1 **Alignment of informative AA sequence regions in fasta format**.
