# Supplementary figures and images for: T3SS-Independent Uptake of the Short-Trip Toxin-Related Recombinant NleC Effector of Enteropathogenic Escherichia coli Leads to NF-κB p65 Cleavage
Source: Front Cell Infect Microbiol. 2017 Apr 13;7:119. doi: 10.3389/fcimb.2017.00119 (PMC5390045; doi:10.3389/fcimb.2017.00119)

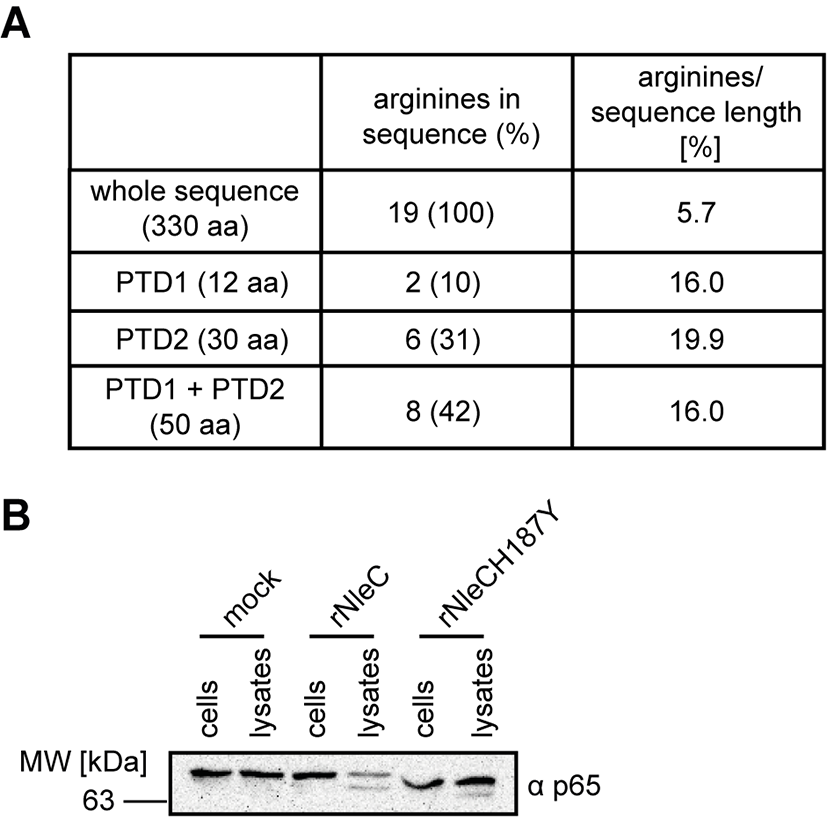

Supplement: Figure S1 — (A) Arginines are enriched in predicted PTDs. The number of arginine residues was counted. Arginine residues per sequence in % (left column) and arginine residues per sequence length in % (right column) were calculated. Arginines are enriched in predicted PTDs. (B) Catalytic activity of rNleCH187Y is not abrogated. HeLa cells or HeLa cell lysates were incubated with 125 μg recombinant proteins (in whole cells this represents 25 μg/ml). Samples were subjected to Western blotting and p65 cleavage was detected using α-p65 antibodies. [file Image1.TIF]

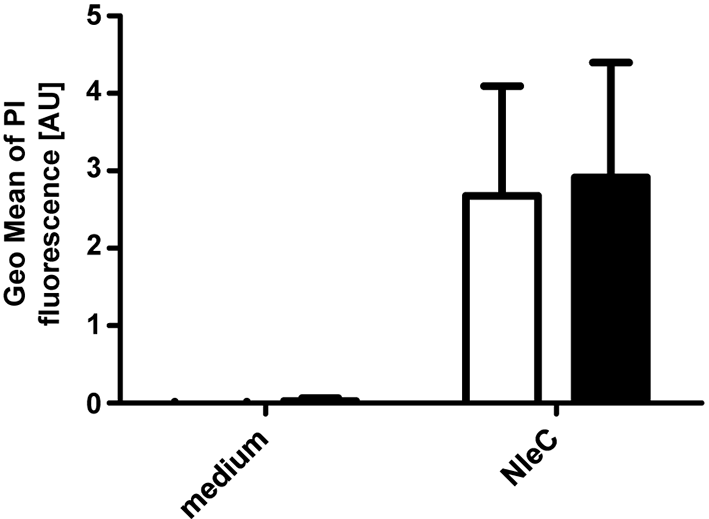

Supplement: Figure S2 — IL1β does not permeabilize cells. HeLa cells were incubated with 50 μg/ml rNleC for 30 min together with 1 μg/ml PI. Cells were stimulated with10 ng/ml IL1β (black bars) for 3.5 h or not (white bars), trypsinized and PI uptake was measured using flow cytometry. The bar graph shows the results of three independent experiments (mean ± SD). [file Image2.TIF]

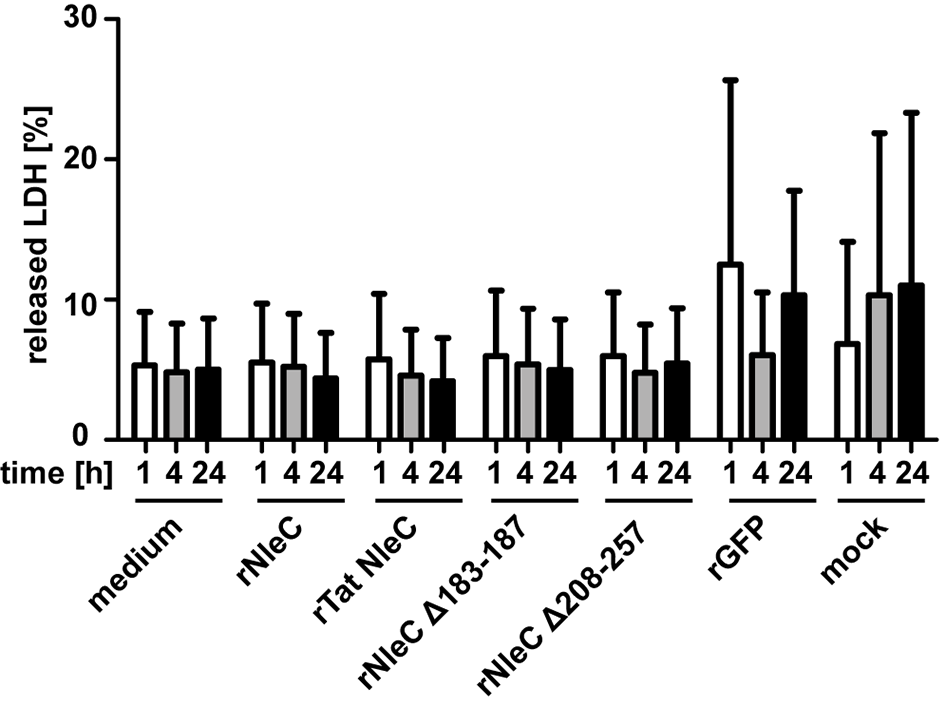

Supplement: Figure S3 — Recombinant proteins are not cytotoxic. The percentage of released LDH was measured to exclude a cytotoxic effect of rNleC or of the rNleC variants on HeLa cells. Experiments are presented as the mean ± SD of three individual experiments. [file Image3.TIF]

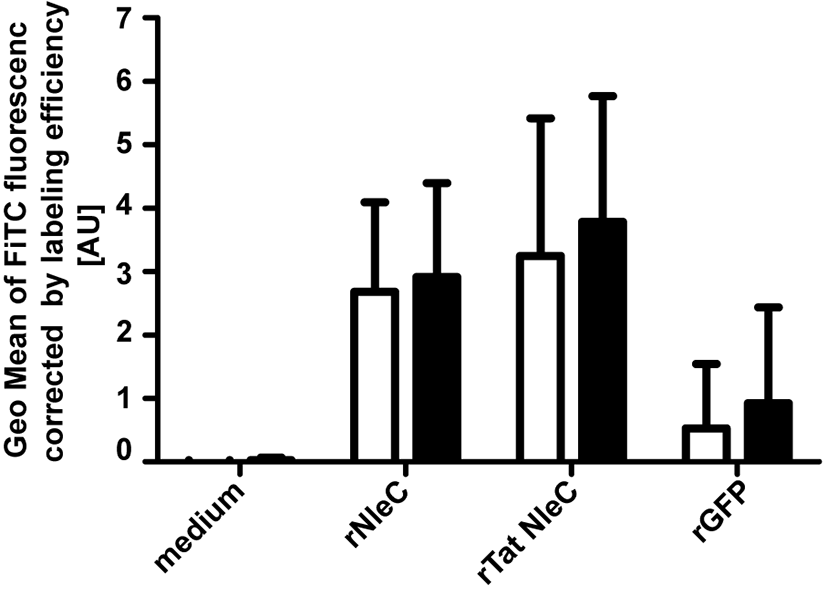

Supplement: Figure S4 — IL1β does not influence the uptake of rNleC or rTat NleC. HeLa cells were incubated with 50 μg/ml protein for 30 min. Cells were stimulated with 10 ng/ml IL1β (black bars) for 3.5 h or not (white bars) and trypsinized. Trypsinized cells were quenched with Trypan Blue (final concentration 0.2%) and fluorescence was subsequently measured by flow cytometry. The bar graph shows the results of three independent experiments (mean ± SD). [file Image4.TIF]

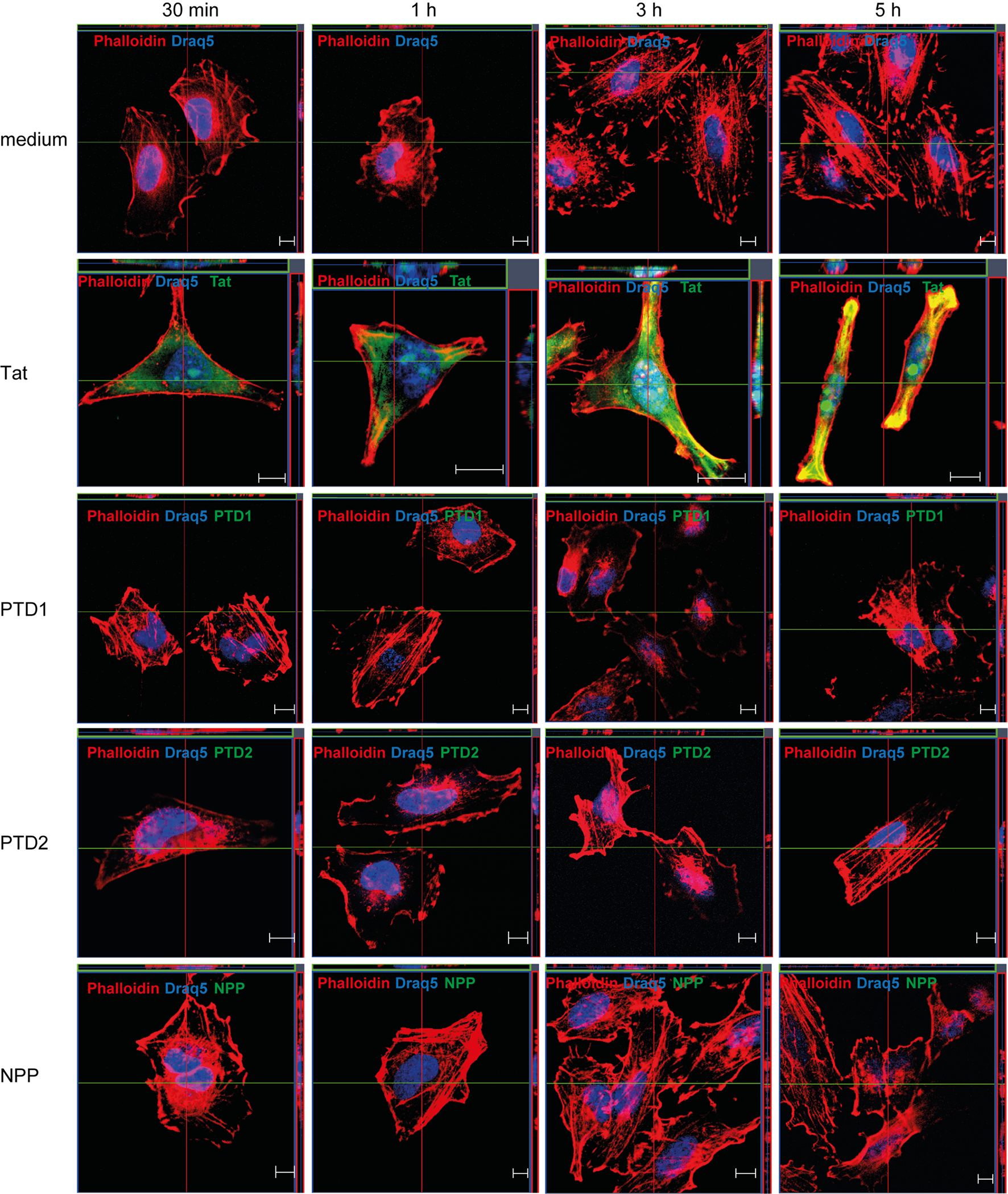

Supplement: Figure S5 — PTD1 and PTD2 are not CPPs. Fluorescence microscopy of HeLa cells incubated with 576 nM FITC-labeled PTD1, PTD2, Tat and NPP (green) for the indicated times. PFA-fixed cells were stained with Phalloidin TRITC (red) and Draq5 (blue). Fluorescence images were generated using a Zeiss LSM 510 microscope. Z-stacks of single cells were taken. [file Image5.TIF]

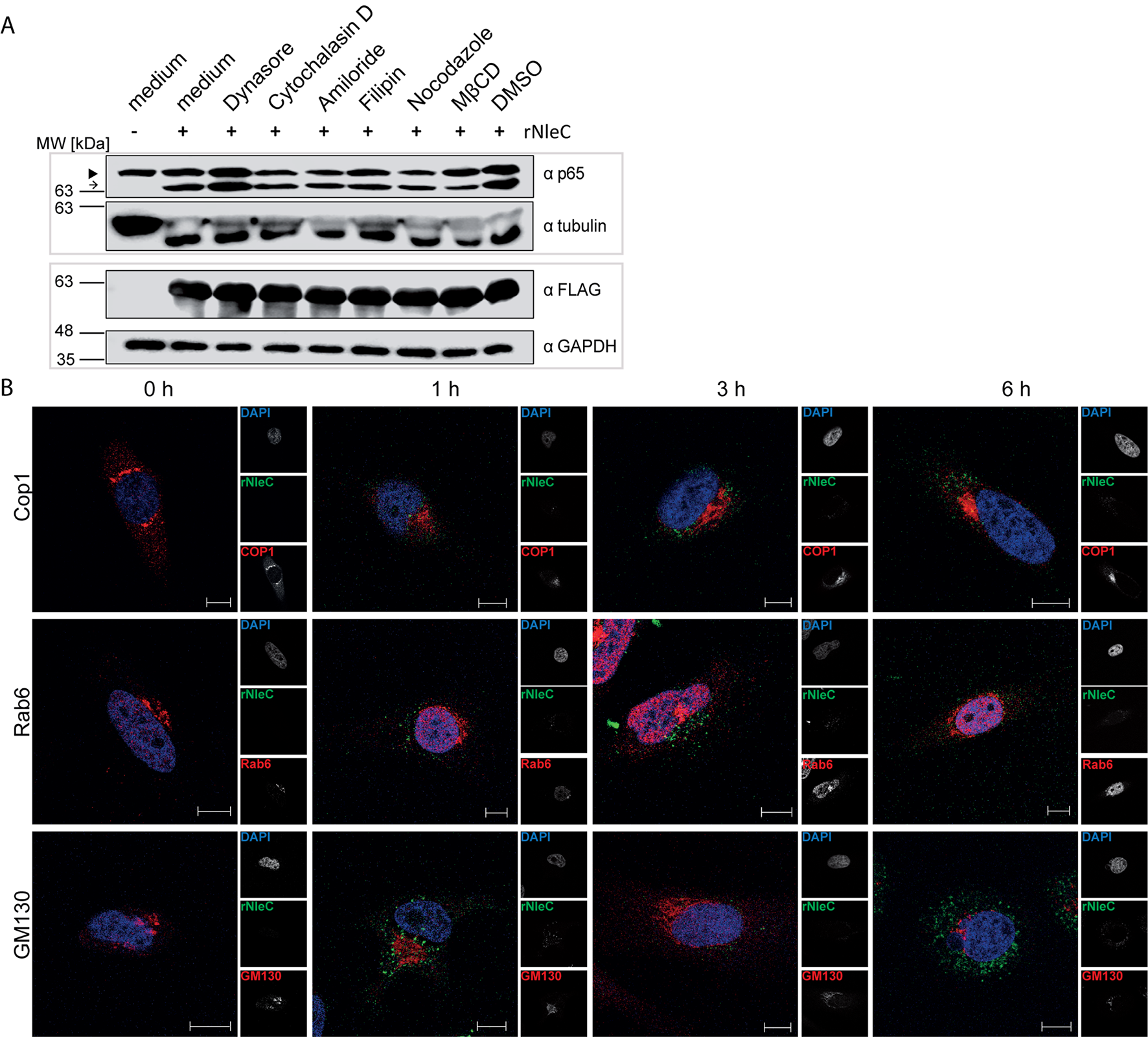

Supplement: Figure S6 — (A) Endocytic inhibitors do not inhibit catalytic activity of rNleC. HeLa cell lysates were incubated with the indicated endocytosis inhibitors (Dynasore 30 mM, Cytochalasin D 200 μM, Amiloride 19 mM, Filipin 3.8 mM, Nocodazole 16.5 mM and MβCD 50 mM) together with rNleC (50 μg/ml) for 30 min at 4°C. Cleavage of p65 was analyzed by Western blotting using α-p65 antibodies. FLAG-tagged rNleC, was detected with an α-FLAG antibody. Tubulin and GAPDH were used as loading controls. gray boxes indicate bands obtained with the same gels. (B) rNleC does not co-localize with markers of retrograde trafficking. HeLa cells were pre-incubated with FITC-labeled rNleC for the indicated times. Cells were fixed, quenched, permeabilized and immunostained with α COP1, α Rab6 or α GM130 antibodies and were visualized with a Cy3-labeled secondary antibody (red). Nuclei were stained with DAPI (blue). Fluorescence images were generated using a Zeiss LSM800 microscope. The scale bar represents 10 μm. [file Image6.TIF]

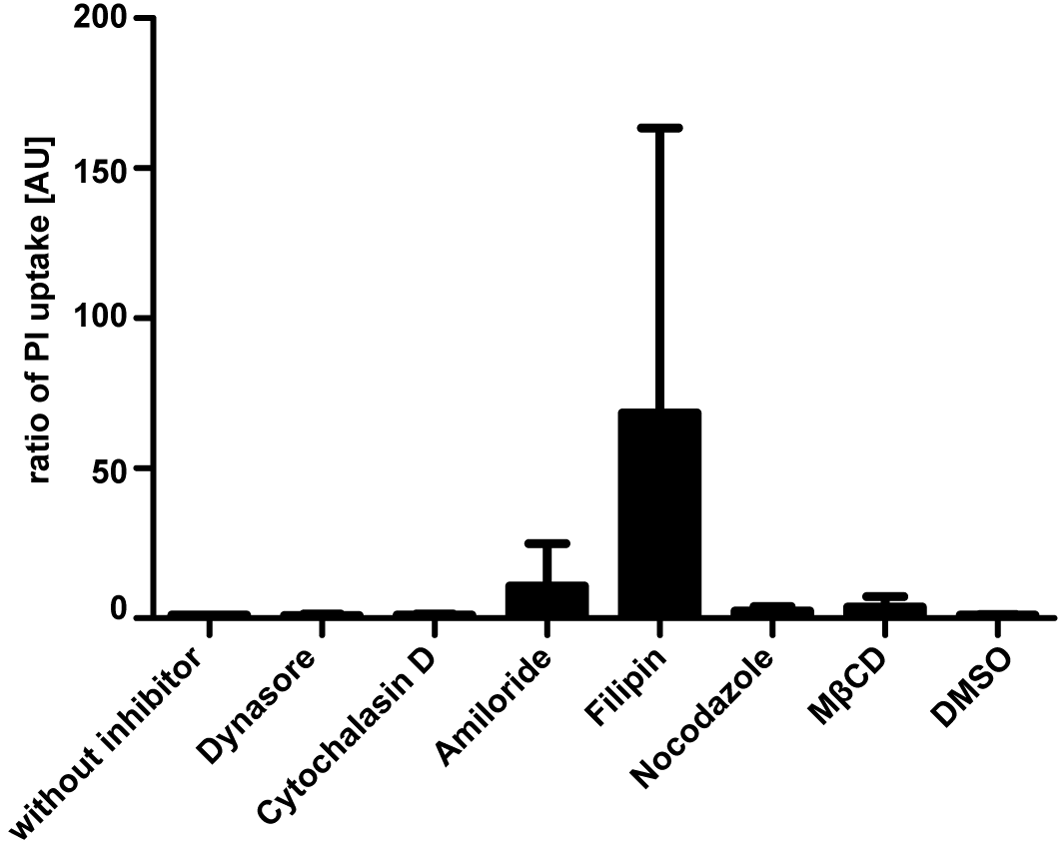

Supplement: Figure S7 — PI uptake of endocytosis inhibitor treated cells. Adherent HeLa cells were pre-incubated with the indicated endocytosis inhibitors for 1 h and treated with FITC-labeled rNleC (50 μg/ml) for 4 h at 37°C. PI fluorescence was subsequently measured by flow cytometry. The bar graph shows the results of at least three independent experiments (mean ± SD). [file Image7.TIF]

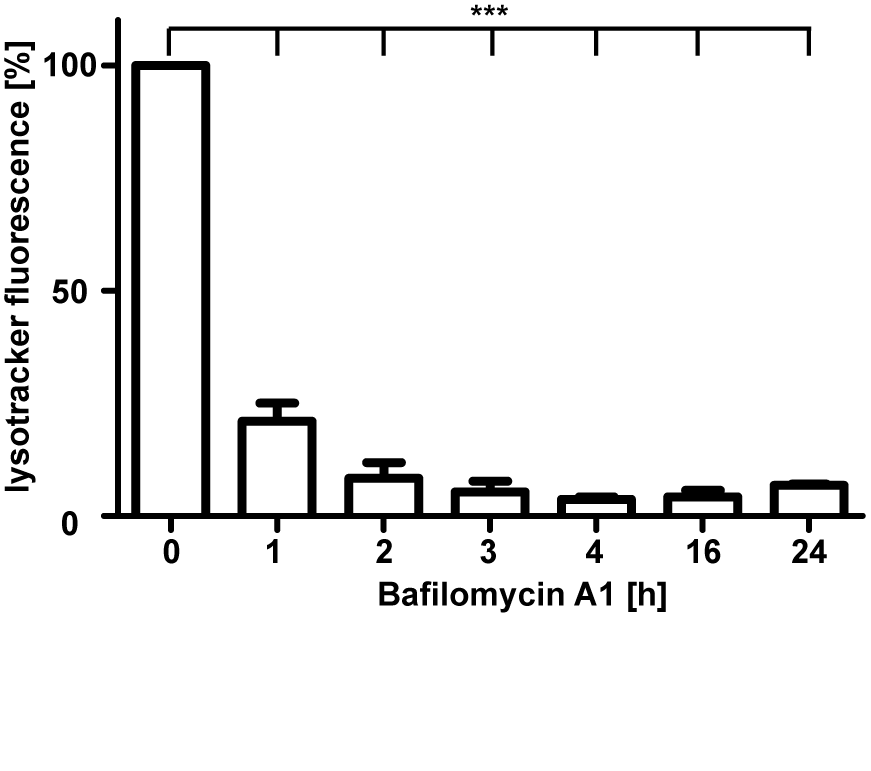

Supplement: Figure S8 — Bafilomycin A1 blocks acidification of lysosomes. HeLa cells were incubated with 10 nM Bafilomycin A1 for the indicated times. 3 h prior to the end of the experiment 400 nM Lysotracker DND99 was added to the supernatant. Cells were trypsinized and the amount of acidified endosomes was measured using flow cytometry. The data are presented after subtraction of background fluorescence and are the mean ± SD of three independent experiments. ***p ≤ 0.001. [file Image8.TIF]

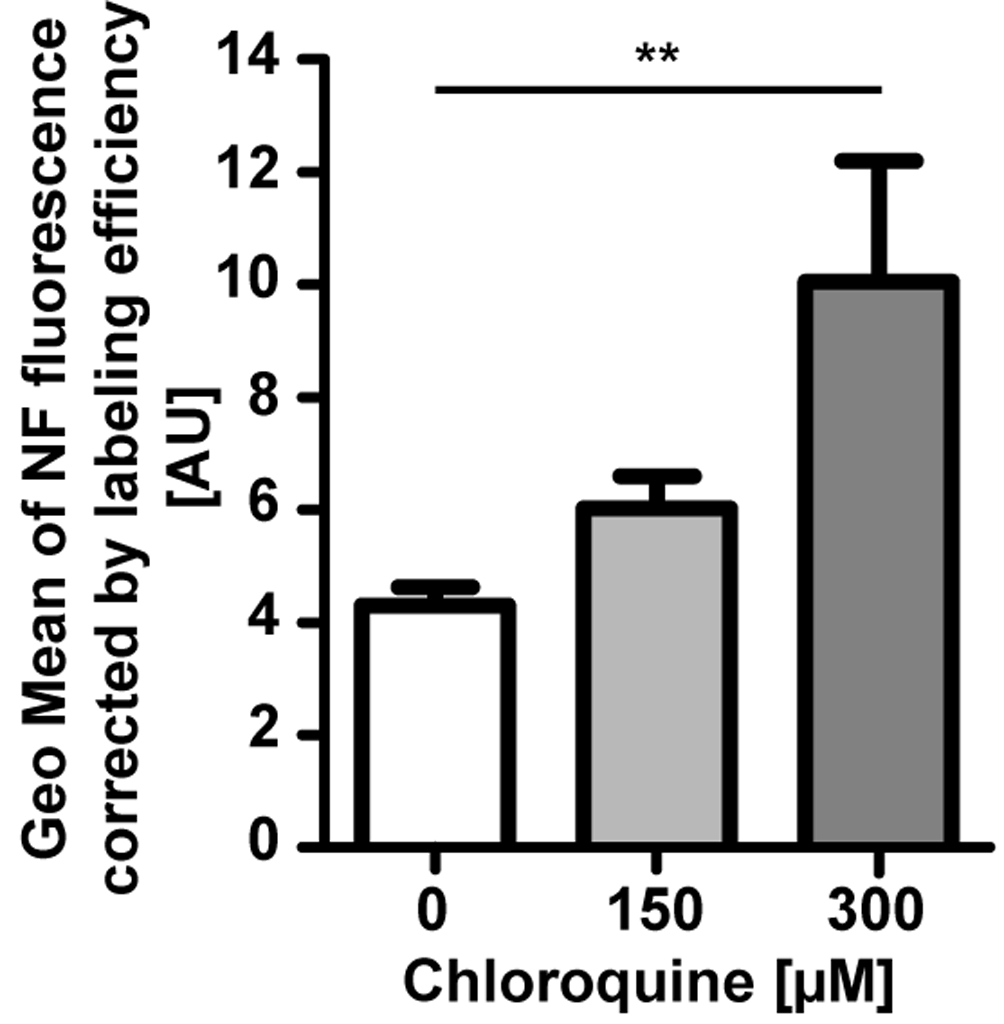

Supplement: Figure S9 — Chloroquine releases endocytosed BSA into the cytosol. HeLa cells were incubated with 0, 150 or 300 μM Chloroquine together with 25 μg/ml BSA for 3 h. Cells were washed and quenched with 0.2 M glycine abrogate signals from the outside of the cell. Geo mean of FL3 fluorescence corrected by labeling efficiency is depicted. Bar graph represents the mean of at least three independent experiments (mean ± SD) with the level of significance indicated (one-way ANOVA, followed by Bonferroni multiple comparison test). **p ≤ 0.01. [file Image9.TIF]

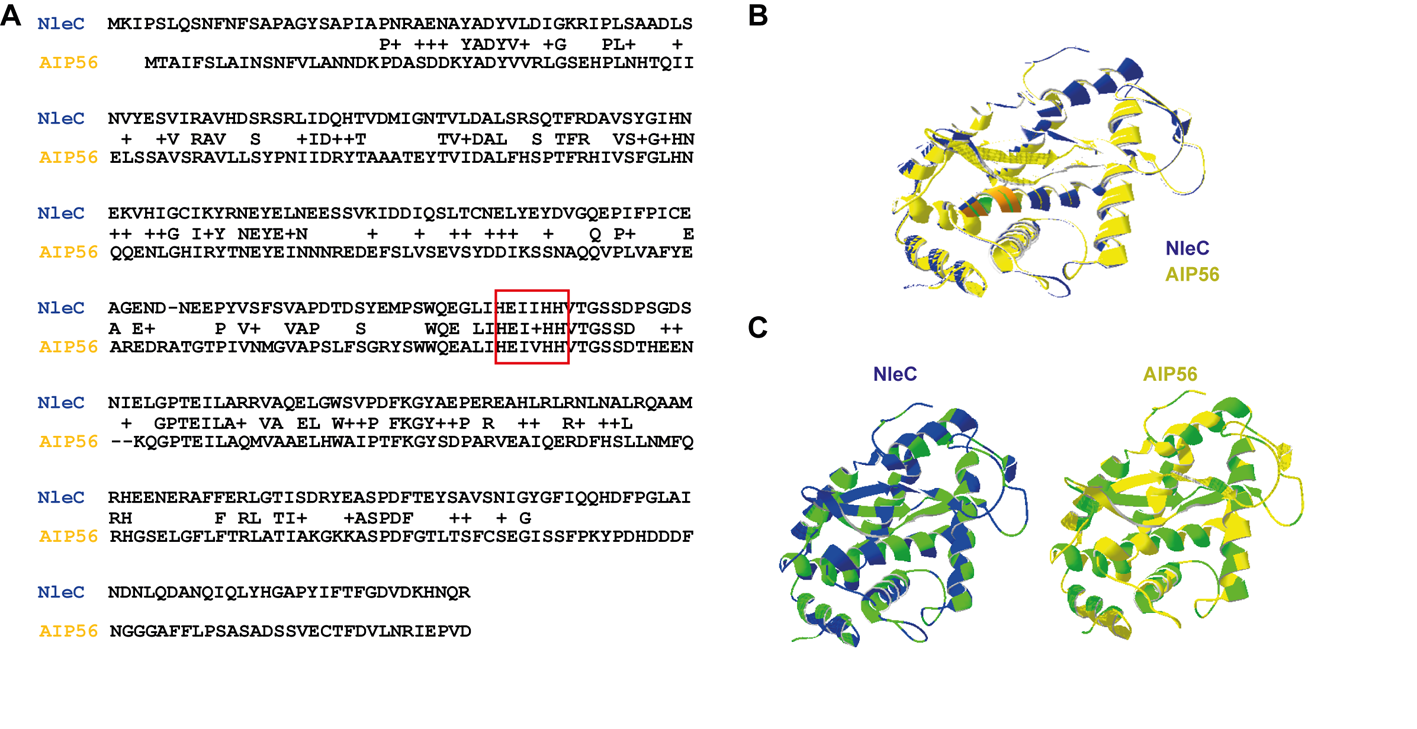

Supplement: Figure S10 — Sequential and structural homologies of NleC and AIP56. (A) Sequence alignment of the A subunit of AIP56 and NleC. (B) Overlay of structure predictions for NleC (blue) and the A subunit of AIP56 (yellow) with Swiss-PdbViewer 4.1.0. The catalytic domain (HEXXH) motif is marked in orange (NleC) or green (AIP56). (C) Hydrophobic amino acids in the sequence of NleC (blue) and the A subunit of AIP56 (yellow) are marked in green using Swiss-PdbViewer 4.1.0. [file Image10.TIF]

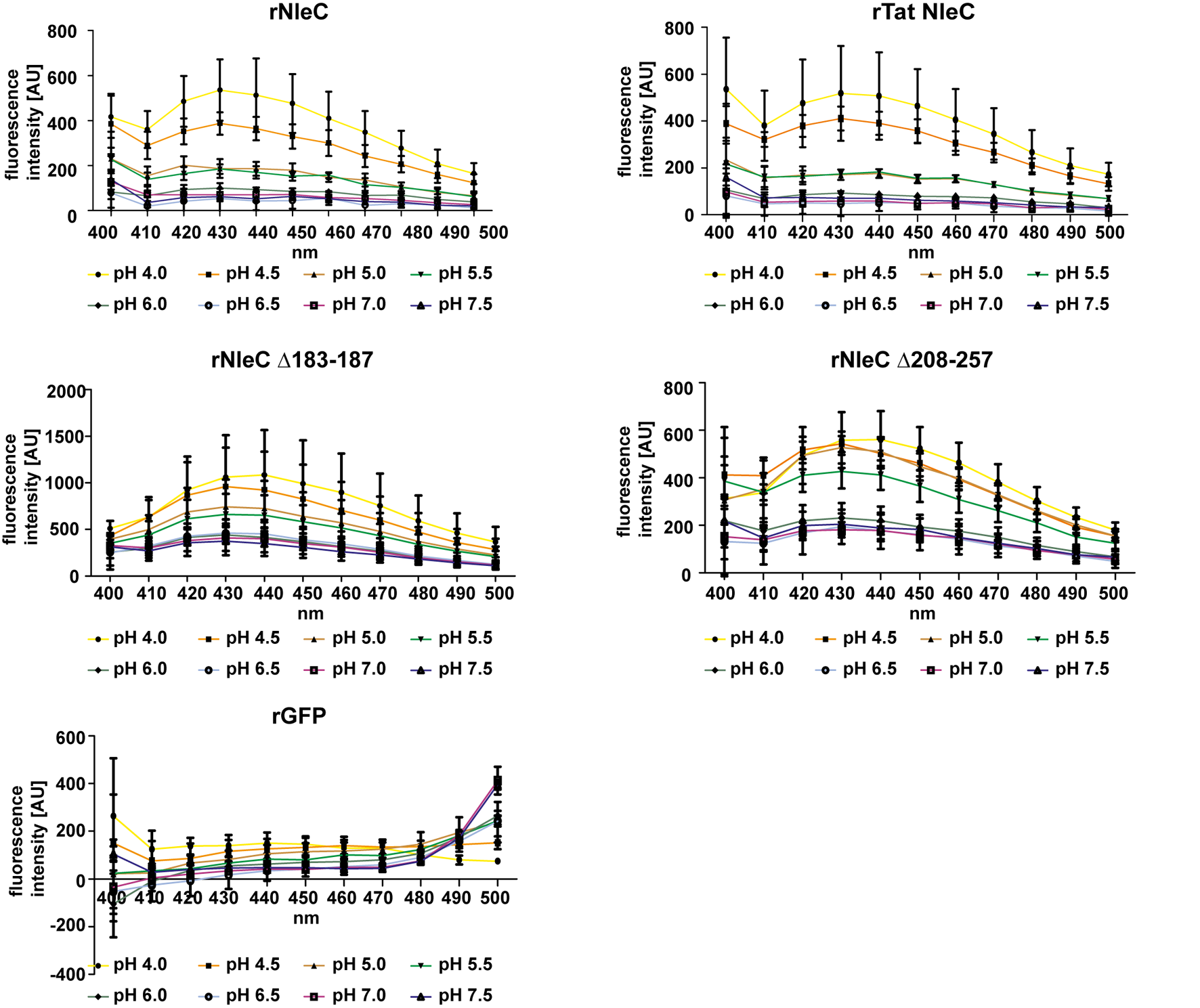

Supplement: Figure S11 — Conformational changes at low pH. Complete spectra of TNS fluorescence of rNleC, rNleC variants and rGFP at pH 7.5, 7.0, 6.5, 6.0, 5.5, 5.0, 4.5, and 4.0. Data are presented after subtraction of background fluorescence and are the mean ± SD of three independent experiments. [file Image11.TIF]
